# Rapid-acting antidepressant drugs modulate affective bias in rats

**DOI:** 10.1126/scitranslmed.adi2403

**Published:** 2024-01-10

**Authors:** Justyna K Hinchcliffe, Sarah A Stuart, Christian M Wood, Julia Bartlett, Katie Kamenish, Roberto Arban, Christopher W Thomas, Aslihan Selimbeyoglu, Shaun Hurley, Bastian Hengerer, Gary Gilmour, Emma S.J. Robinson

**Affiliations:** 1 University of Bristol, School of Physiology, Pharmacology and Neuroscience, Biomedical Sciences Building, Bristol, BS8 1TD, UK; 2 Department of Physiology, Development and Neuroscience, University of Cambridge, Cambridge, CB3 2DY, UK; 3 CNS Diseases Research, Boehringer Ingelheim GmbH & Co. KG, Biberach an der Riss, Germany; 4 COMPASS Pathways plc, London, W1F 0DQ, UK

## Abstract

How rapid-acting antidepressants (RAADs), such as ketamine, induce immediate and sustained improvements in mood in patients with major depressive disorder (MDD) is poorly understood. A core feature of MDD is the prevalence of cognitive processing biases associated with negative affective states, and the alleviation of negative affective biases may be an index of response to drug treatment. Here, we used an affective bias behavioral test in rats, based on an associative learning task, to investigate the effects of RAADs. To generate an affective bias, animals learned to associate two different digging substrates with a food reward in the presence or absence of an affective state manipulation. A choice between the two reward-associated digging substrates was used to quantify the affective bias generated. Acute treatment with the RAADs ketamine, scopolamine or psilocybin selectively attenuated a negative affective bias in the affective bias test. Low, but not high, doses of ketamine and psilocybin reversed the valence of the negative affective bias 24 hours after RAAD treatment. Only psilocybin, but not ketamine or scopolamine, led to a positive affective bias that was dependent on new learning and memory formation. The re-learning effects of ketamine were dependent on protein synthesis localised to the rat medial prefrontal cortex and could be modulated by cue-reactivation, consistent with experience-dependent neural plasticity. These findings suggest a neuropsychological mechanism that may explain both the acute and sustained effects of RAADs, potentially linking their effects on neural plasticity with affective bias modulation in a rodent model.

## Introduction

Major depressive disorder (MDD) is estimated to affect more than 300 million people globally with a marked increase in prevalence due to the COVID-19 pandemic (1). In 2000, Berman et al. showed that the NMDA receptor antagonist, ketamine, induced rapid and sustained antidepressant effects in a treatment-resistant cohort with MDD after a single dose (2). Since the discovery of ketamine as a rapid-acting antidepressant (RAAD), other pharmacologically unrelated compounds have also been shown to have rapid and sustained antidepressant effects in clinical populations (3–5). Despite more than two decades of clinical and preclinical research, however, the mechanisms that underlie the effects of RAADs on mood remain to be fully elucidated. Furthermore, how these pharmacologically distinct treatments converge to improve the psychological symptoms of MDD has not yet been explained.

Modelling clinically relevant symptoms of MDD in animals is key to understanding the relationships between the biological and experience-dependent factors that drive the behavioral symptoms of depression and its treatment. A core feature of MDD is the prevalence of cognitive processing biases associated with negative affective states, termed negative affective biases (6–9), which may be a key factor underpinning low mood and negative rumination (10). In this neuropsychological model of depression, negative affective biases play a causal role in vulnerability, precipitation, and maintenance of MDD (10). Alleviation of negative affective bias may be an index of responsivity to pharmacological and psychological treatments (11–13). We and others have previously suggested that latency to the alleviation of negative affective biases contributes to the speed of onset of antidepressant action (11, 14). We have developed a rodent affective bias test based on an associative learning task where the animals learn to associate a particular digging substrate with a food reward. In a series of pharmacological, neural circuit and phenotypic studies, we have established validity of this digging task (14–17). This task has been used to quantify positive or negative affective state-induced biases generated when treatments are given before the associative learning sessions. This task also has been used to test how treatments modulate retrieval of negatively biased memories by first generating a negative affective bias and then administering RAAD treatment shortly before (acute) or 24 hours before (sustained) performing a choice test. Although the pharmacological characterization of human affective bias modification is less extensive than we have established in this rodent affective bias test, there is a high degree of similarity observed between acute drug effects in our rodent assay and in studies of healthy human volunteers (12, 16). This includes similar findings for conventional antidepressants, a lack of efficacy of the failed antidepressant aprepitant, and induction of negative affective biases after treatment with a drug inducing depression, rimonabant.

Here, we sought to explore the neuropsychological mechanisms underlying the effects of three pharmacologically distinct RAADs. We first compared the effects of ketamine (a NMDA antagonist), scopolamine (a muscarinic antagonist) and psilocybin (a serotonergic psychedelic) using three different affective bias test protocols in rats. We also used a control memory retrieval test, the reward learning assay, to establish the specificity of any affective bias modifications. To explore the underlying mechanisms, we directly infused ketamine into the rat medial prefrontal cortex, a key brain region implicated in MDD (14, 18, 19), and analyzed the effects of RAAD treatment (20, 21). We then explored the role of protein synthesis-dependent and independent mechanisms in acute versus sustained modulation of affective biases and used cue-reactivation to investigate the interaction between the effects of neural plasticity and experience-dependent learning and memory.

## Results

### Affective bias test and reward learning assay in rats

The affective bias test involved each animal learning two independent substrate-reward associations under either RAAD treatment or control conditions (figure S1, movie S1). The specific digging substrates provided cues which the animals learned were predictive of finding a food reward (table S1). Each animal learned two independent substrate-reward associations over four pairing sessions with treatment/control, substrate and order of presentation fully counter-balanced (table S2A-C). The value of the food reward was kept constant throughout. The affective bias generated was quantified using a choice test where the animals were presented with the two previous reward-associated digging substrates at the same time and their choices over 30 randomly reinforced trials were recorded (figure S1). To investigate modulation of a negatively biased memory, we first generated a negative affective bias using the benzodiazepine inverse agonist FG7142 (3mg/kg) or corticosterone (10mg/kg) and then administered the RAAD 1 hour or 24 hours before the choice test (table S3). We also tested whether RAADs would directly induce an affective bias by administering the RAAD before one of the substrate-reward association tests. To determine if the effects of RAAD treatment were specific to an affective state-induced bias, we also used a second learning and memory test as a control. Following the same basic protocol as the affective bias test, two different digging substrates were independently paired with a food reward. In this test, one substrate was paired with a higher value reward (two food pellets instead of one food pellet) leading to a reward-induced bias during the subsequent choice test.

### RAAD treatment attenuates memory-specific negative affective bias in rats

**Figure 1:**
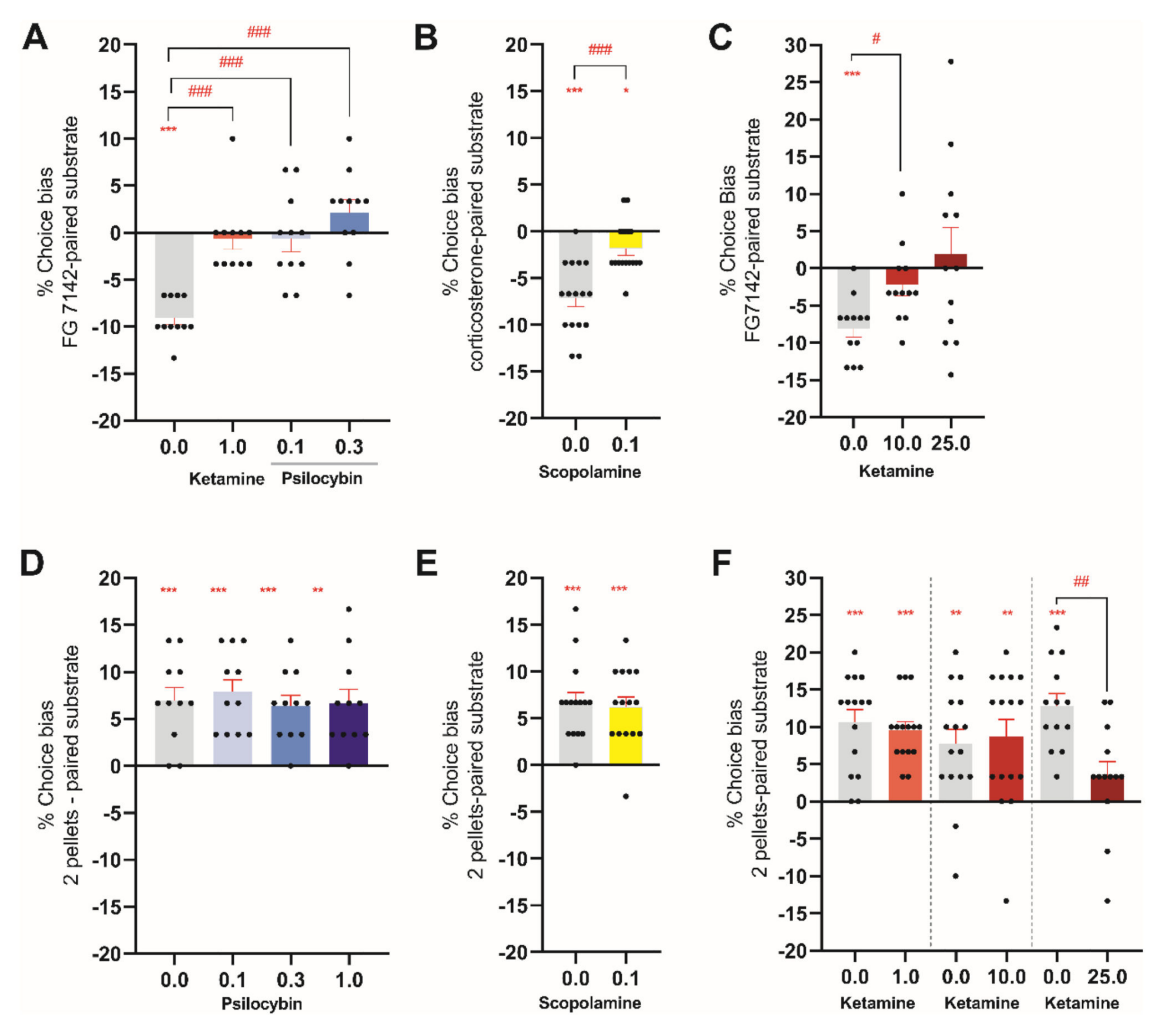
Low but not high RAAD doses attenuate negative affective bias in rats. Male rats were subjected to both the affective bias test and reward learning assay, bowl-digging tasks where rats learned to associate a specific digging substrate with recovery of a food reward. (**A, B**) Following induction of a negative affective bias with FG7142 or corticosterone, male rats were injected with low dose ketamine (1mg/kg; n=12) or psilocybin (0.1 and 0.3mg/kg; n=12) (**A**) or scopolamine (0.1mg/kg; n=12) (**B**). The animals were then subjected to a choice test involving choosing between two reward-associated digging substrates. (**C**) To test the effects of different doses of ketamine, a negative bias was first induced and the rats were then administered ketamine (10 or 25mg/kg; n=12) prior to administration of the choice test. (**D-F**) In the reward learning assay, a reward-associated positive bias was induced using high (two pellets) versus low (one pellet) reward pairing sessions followed by administration of psilocybin (0.1, 0.3 and 1.0mg/kg; n=12) (**D**) or scopolamine (0.1mg/kg; n=12) (**E**) or ketamine (1, 10 or 25mg/kg; n=12) (**F**) before administration of the choice test. Data are shown as mean % choice bias ± SEM (bars) as well as individual data points (dots, n=11-16). Data were analyzed with one sample t-test against a null hypothesis mean of 0% choice bias (*p<0.05, **p<0.01, ***p<0.001) and pairwise comparisons were done using paired t-test following main effect in ANOVA (##p<0.01, ###p<0.001).

We first generated a memory specific, negative affective bias in rats with either the benzodiazepine inverse agonist, FG7142 (3mg/kg), or the stress hormone corticosterone (10mg/kg) (figure S2). The doses for corticosterone and FG7142 were chosen based on dose-response studies carried out previously in rats administered the affective bias test (16, 22). During the choice test between the two reward-associated digging substrates, rats receiving vehicle made fewer choices for the treatment-paired digging substrate consistent with a negatively biased memory (one-sample t-test, p<0.0001, Fig. 1A-C). Acute psilocybin or ketamine, administered prior to the choice test, attenuated the negative bias (repeated measures ANOVA, F3,30=19.94, p<0.0001, *η*^2^=0.67, n=11, with Dunnett’s test, p<0.05) (Fig. 1A). A negative bias was also attenuated when animals received the higher dose of psilocybin 1.0mg/kg (figure S3A) or scopolamine (0.1mg/kg, two-tailed paired t-test: t15=5.168, p=0.0001 vs vehicle, *d*=1.29, n=16) (Figure 1B). There was no evidence of non-specific impairments for any of the treatments or doses tested (tables S4, S5). During the choice test, psilocybin increased “wet dog” shake behavior at 0.3mg/kg (table S6), and increased both wet dog shakes and head twitch responses at 1mg/kg (table S7). Ketamine has only been found to have antidepressant effects at low doses (23–25), so we also tested higher doses of ketamine (10mg/kg, 25mg/kg; n=12) (Fig. 1C). Although both the mid and high doses of ketamine (10mg/kg, 25mg/kg) attenuated the negative bias (Fig. 1C), they increased omitted trials and latency to respond indicating non-specific effects (figure S4 and table S8).

### Effect of low dose RAADs on negative affective bias is not due to impaired learning and memory

The attenuation of the bias to ~0% could result from a generalised amnesic effect so we used the reward learning assay to establish specificity for an affective state-induced memory bias (figure S5). The reward learning assay has the advantage of using an almost identical protocol to the affective bias test, but the rats remain in the same affective state throughout and a bias is generated by changing the absolute value of the reward. Animals treated with low dose ketamine, psilocybin or scopolamine expressed similar reward-induced positive biases compared to the vehicle control, confirming that the affective bias modulation by RAADs was specific (Figure 1D-F). Although all ketamine doses attenuated a negative bias in the affective bias test, there was an impairment in the reward learning assay for 25mg/kg ketamine, suggesting a non-specific effect on memory (Figure 1F). Both 10 mg/kg and 25mg/kg doses of ketamine also increased omissions and latencies during the reward learning assay followed by the choice test (table S8). Psilocybin increased wet dog shakes at a dose of 0.3mg/kg and both wet dog shakes and head twitch responses at 1mg/kg during the choice test (table S9).

### RAAD-induced modulation of negative affective bias is sustained for 24 hours

**Figure 2:**
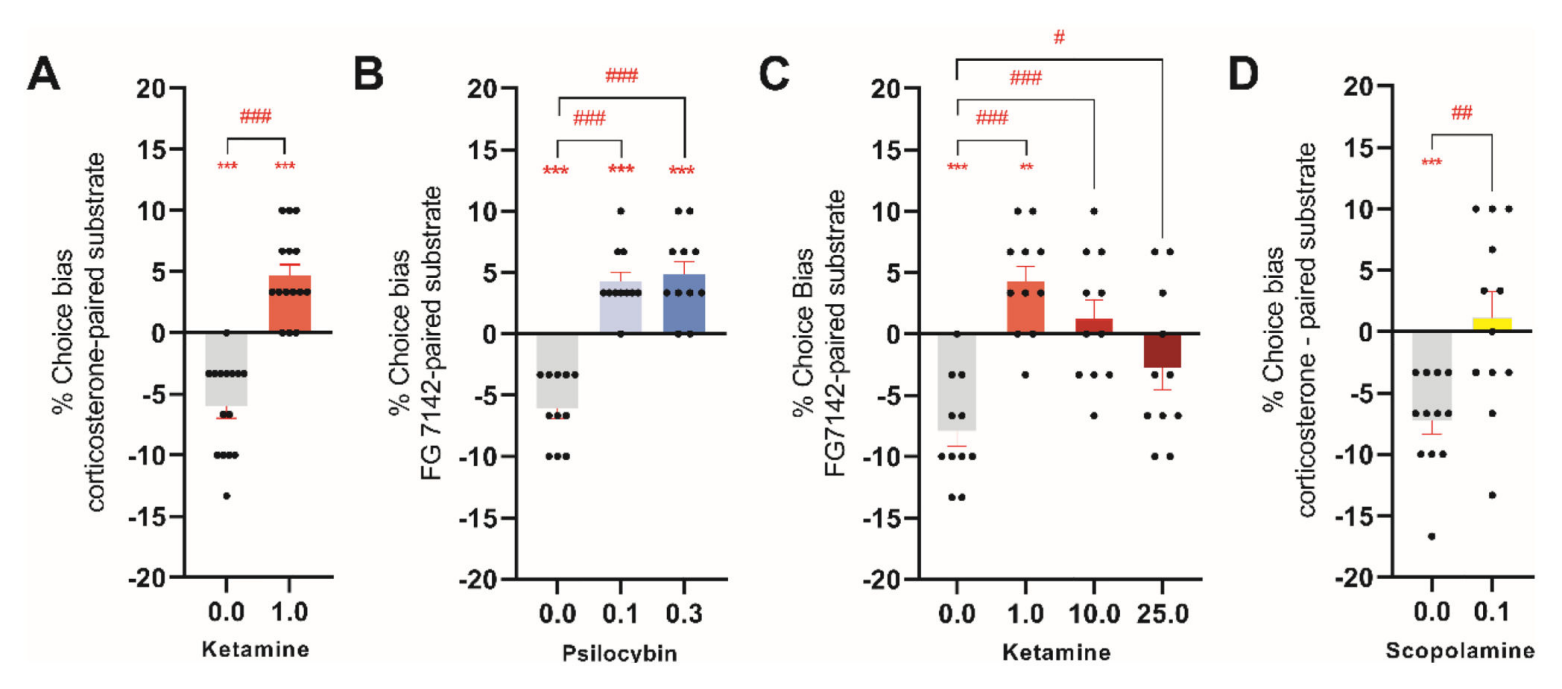
Inversion of a negative bias 24 hours after low dose ketamine or psilocybin. A negative affective bias was first induced in rats using FG7142 or corticosterone during the pairing sessions of the affective bias test. The RAAD was then administered by ip injection 24 hours before the choice test. (**A, B**) Animals were treated with low dose ketamine (1.0mg/kg; n=15) (**A**) or psilocybin (0.1, 0.3 mg/kg; n=11) (**B**) and were subjected to the choice test 24 hours after treatment. (**C, D**) Animals were treated with higher doses of ketamine (10 or 25 mg/kg, n=11) (**C**) or scopolamine (0.1 mg/kg; n=12) (**D**) and were subjected to the choice test 24 hours after treatment. Data are shown as mean % choice bias ± SEM (bars) as well as individual data points (symbols). Data were analyzed with a one sample t-test against a null hypothesis mean of 0% choice bias (**p<0.01, ***p<0.001) and pairwise comparisons were done using a paired t-test following main effect in ANOVA (#p<0.05, ##p<0.01, ###p<0.001).

A challenging aspect of relating the pharmacology and underlying mechanisms of action of RAADs to their clinical benefits is that the effects are sustained long after the drug has been eliminated from the body. Animal studies suggest these prolonged effects are mediated by neural plasticity (26, 27). We tested whether the modulation of negative affective bias by RAADs could be sustained for at least 24 hours due to circuit-specific changes in neural plasticity. First, we generated a negative affective bias and then administered one of three RAADs 24 hours before the choice test (Figure S6). We observed a consistent negative bias in our vehicle-treated group showing that the biased memory was retained over this time frame (one sample t-test, p<0.05, Figure 2A-D). Unexpectedly, low dose ketamine led to a positive affective bias in this test indicating re-learning with a positive affective valence (one sample t-test: t14=5.137, p=0.0002, two-tailed paired t-test: t14=8.702, p=0.0001, *d*=2.25, n=15 and F3,30=12.79, p<0.0001, *η*^2^=0.56, n=11) (Figure 2A, C). The same was found for psilocybin treatment (repeated measures ANOVA: F3,30=27.16, p<0.0001, *η*^2^=0.81, n=11) (Figure 2B). Scopolamine treatment (two-tailed paired t-test: t11=4.022, p=0.0020, *d*=1.16, n=12) (Figure 2D), and higher doses of ketamine (Figure 2C) and psilocybin (figure S3B) ameliorated the negative affective bias at the 24 hour timepoint, but did not induce any positive affective bias. No treatment impacted response latency during choice tests (table S10).

### Psilocybin positively biases new reward memories

**Figure 3:**
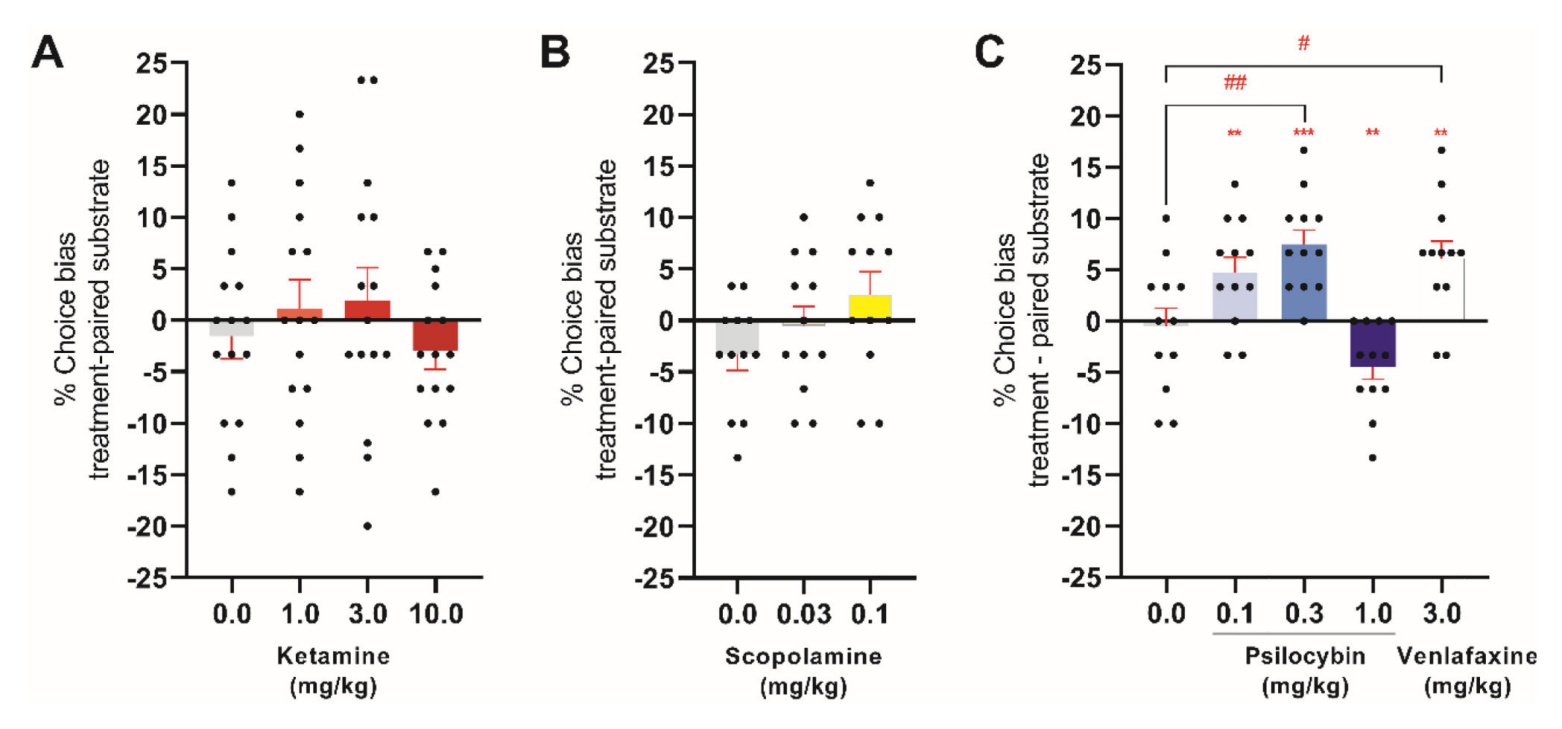
RAAD treatment induces a positive affective bias associated with new learning and memory. To test the effects of RAAD treatment on learning and new memories, rats were treated with the RAAD before the pairing sessions, with the choice test performed 24 hours after the last pairing session. Rats were acutely administered doses of ketamine (1.0, 3.0, 10.0 mg/kg; n=15) (**A**) or scopolamine (0.03, 0.1 mg/kg; n=12 (**B**) and were subjected to the affective bias test immediately after drug treatment. (**C**) Rats were acutely administered doses of psilocybin (0.1mg/kg or 0.3mg/kg, n=12) or the antidepressant drug venlafaxine (3.0mg/kg, n=12) and were subjected to the affective bias test 1 hour after drug treatment. Only venlafaxine (p=0.0117) and psilocybin 0.3mg/kg (p=0.0019) were significantly different from the vehicle control group. Data are shown as mean % choice bias ± SEM (bars) as well as individual data points (symbols). Data were analyzed with a one sample t-test against a null hypothesis mean of 0% choice bias (**p<0.01, ***p<0.001) and Dunnett’s test (#p<0.05, ##p<0.01).

Conventional antidepressant drugs induce a positive affective bias in humans (12) and rats (16), but fail to modify biased memories. These neuropsychological effects are hypothesized to be due to interactions between the biological effects of the drug and environmental factors, which may explain why the subjective effects of these drugs on mood are delayed (28). We examined whether RAADs could positively bias new memories in rats by administering the RAADs before one of the substrate-reward pairing sessions and with the choice test carried out 24 hours after the last RAAD treatment (figure S7). Low dose psilocybin induced a positive affective bias similar to the conventional antidepressant venlafaxine (repeated measures ANOVA F4,44=10.93, p<0.0001, *η*^2^=0.50, n=12) (Figure 3C), whereas neither ketamine nor scopolamine treatment positively biased new learning (Figure 3A,B). High dose psilocybin and scopolamine resulted in slower latency to approach the bowl and dig in the substrate compared to vehicle (table S11). Psilocybin induced behavioral correlates of psychedelic activity (table S12), and at a high dose induced a negative affective bias (Figure 3C).

### Ketamine’s effect on learning is mediated by rat medial prefrontal cortex neural plasticity

**Figure 4:**
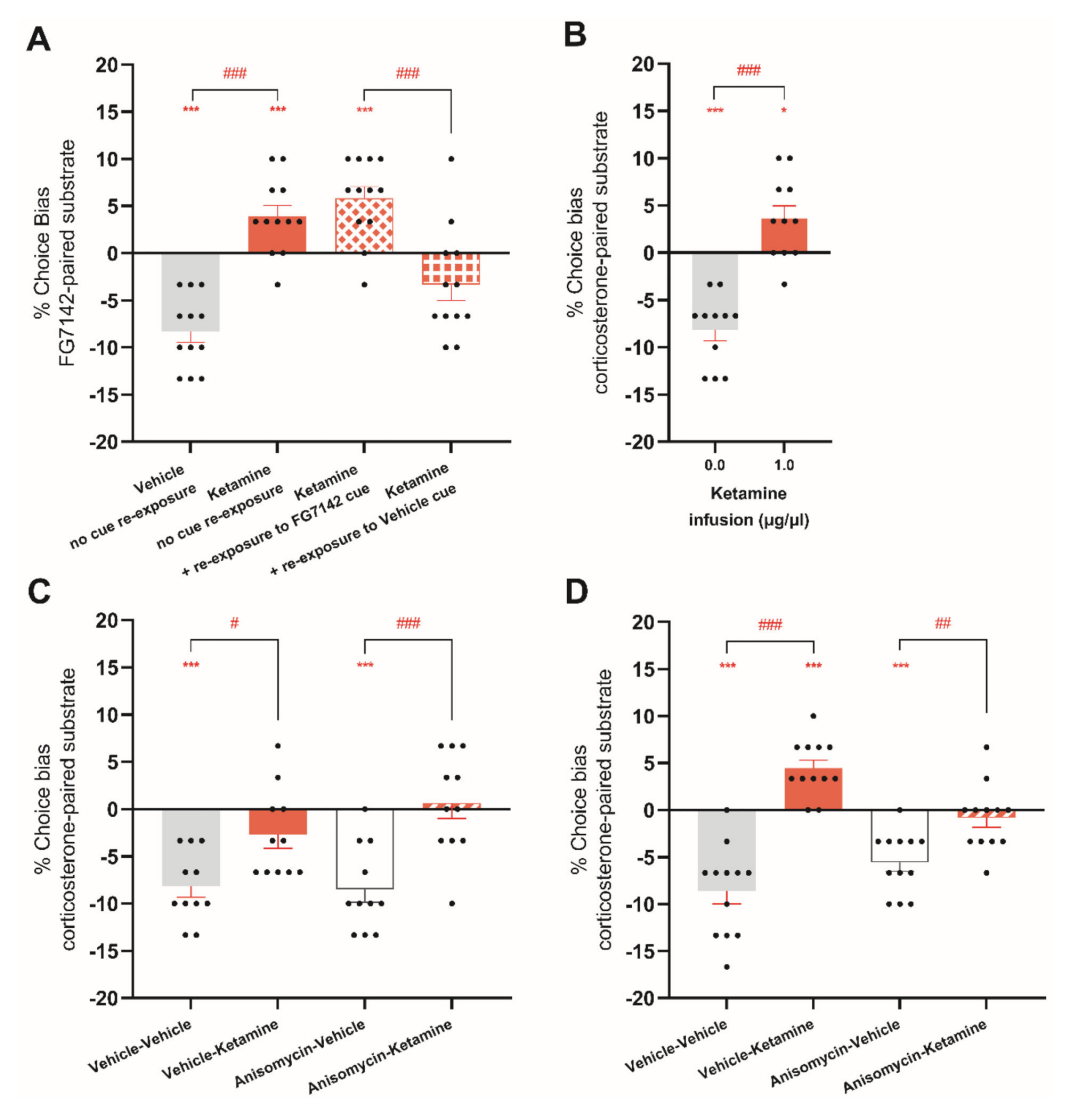
Experience-dependent neural plasticity underlies the re-learning effect 24 hours after RAAD treatment. (**A**) Rats were treated with ketamine and then 24 hours later were subjected to the affective bias test with or without re-exposure to the cue learned during paired training sessions after FG7142 treatment (n=12). (**B**) Following induction of a negative affective bias with corticosterone, male rats were subjected to direct infusion of ketamine into the medial prefrontal cortex (1.0 µg/µl; n=11) and then 24 hours later were subjected to the choice test. (**C, D**) Inversion of the negative affective bias 24 hours after systemic ketamine dosing (1.0 mg/kg; n=12, t= -24 hours) (**D**) but not after acute ketamine dosing (1.0 mg/kg; n=11, t= -1 hour) (**C**) was protein synthesis dependent as shown by medial prefrontal cortex infusion of anisomycin (100ul/ug) or vehicle control. Data are shown as mean % choice bias ± SEM (bars) as well as individual data points (symbols). Data were analyzed with a one sample t-test against a null hypothesis mean of 0% choice bias (**p<0.01, ***p<0.001) and a paired t-test with value adjusted for the number of comparisons (#p<0.05, ##p<0.01, ###p<0.001).

To examine the mechanisms underlying affective bias modification, we investigated ketamine treatment further. To test the hypothesis that negatively biased memories were more likely to be spontaneously re-activated and hence lead to the observed inversion of the affective bias 24 hours after ketamine treatment, we used a cue re-activation test. Animals were dosed with ketamine or vehicle one hour before being briefly re-presented with either the neutral-paired or negative affective state-paired digging substrate cue (Figure S8). Cue presentation lasted only ~3 seconds and occurred over a single trial without reinforcement, after which animals were returned immediately to the home cage for 24 hours before the choice test was administered. Consistent with our predictions, a similar positive affective bias was observed for animals with either no cue re-activation or after exposure to the digging substrate cue associated with FG7142 (Fig. 4A). However, exposure to the cue learned during the neutral state (no FG7142) attenuated the positive bias (paired t-test, t11=4.457, p<0.01) with most animals now exhibiting a bias towards the neutral-paired substrate (Figure 4A). This result indicated that ketamine’s effects 24 hours after dosing could be experience-dependent involving memory re-activation and re-learning. To ensure that these findings were not due to a recency bias (i.e. animals making a choice based on their most recent substrate-reward pairing), we also analysed the data based on the cue used in the last pairing session and found no evidence to suggest that this was the main factor resulting in the effects observed (figure S9).

We previously found that acute infusions of ketamine (14) or the 5-HT_2A_ agonist 2,5-dimethoxy-4-iodoamphetamine in the rat medial prefrontal cortex attenuated a negative affective bias (figure S10). We tested whether a ketamine infusion (1µg/µl) into the rat medial prefrontal cortex was sufficient to replicate the effects observed 24 hours after systemic ketamine administration. Cannula placements were verified using postmortem histology and all animals were included in the analysis (figure S11). Similar to systemic ketamine dosing, ketamine infusions into the medial prefrontal cortex resulted in attenuation of the negative bias relative to infusion of vehicle (two-tailed paired t-test: t10=8.168, p=0.0001 vs vehicle, *d*=2.46, n=11) and reversal to a positive affective bias (one sample t-test: t10=2.782, p=0.0194) (Figure 4B). We next used infusions of the protein synthesis inhibitor anisomycin delivered into the rat medial prefrontal cortex 30 minutes before systemic ketamine dosing, to investigate the role of protein synthesis-dependent mechanisms 1 hour after and 24 hours after ketamine dosing. Anisomycin did not show an effect on the acute (1 hour) ketamine-induce attenuation of a corticosterone-induced negative affective bias (repeated measures ANOVA main effect of ketamine F1,10=20.426, p=0.001, *η*^2^=0.03, n=11) (Figure 4C). There was no effect of anisomycin (F1,10=0.883, p=0.369) and although there was a ketamine-anisomycin interaction (F1,10=8.780, p=0.014, *η*^2^=0.50), post-hoc pairwise comparisons found no evidence that inhibition of protein synthesis prevented the acute effects of ketamine. In contrast, anisomycin infusions into the rat medial prefrontal cortex blocked the formation of a positive affective bias 24 hours after systemic ketamine treatment, but had no effect on the attenuation of the negative affective bias, suggesting different underlying mechanisms (main effect of systemic treatment repeated measures ANOVA, F1,11=44.346, p=0.0001, *η*^2^=0.80, n=12 and systemic x infusion interaction repeated measures ANOVA, F1,11=16.176, p=0.002, *η*^2^=0.60) (Figure 4D). There were no effects of treatment on latency during the choice test (table S13).

## Discussion

Here, we provide evidence that RAADs could mediate their effects on mood through their ability to induce acute and sustained modulation of negative affective biases, a core feature of MDD (3, 6–9). Our findings reveal that affective bias modification in rats is a neuropsychological mechanism shared by RAADs and could explain how their acute pharmacological effects lead to sustained clinical benefits. We also identified differences in the specific way each RAAD interacts with affective biases, which aligns with their clinical efficacy and particularly with their duration of benefits. Based on these findings, we suggest that the long duration of clinical benefit seen with psilocybin (3, 5) could arise from its ability to modulate negatively biased memories and to facilitate positively biased learning of both past and new memories. We also propose that RAADs, at doses that impair learning and memory, are less effective because they either lack the ability to facilitate positively biased re-learning e.g. scopolamine or have a narrow dose range when these selective effects occur e.g. ketamine. We also propose that our affective bias test data supports a two-phase neuropsychological model of affective bias modification. In the first phase, circuits in the medial prefrontal cortex that generate affective biases are selectively modulated, leading to rapid and sustained attenuation of negative affective biases. In the second phase, the therapeutic window generated in phase 1 permits memories to be retrieved and re-learned with a more positive valence that sustains the beneficial effects on mood (figure S12).

Ketamine, psilocybin and scopolamine target different receptors but there is convergence in their effects at the cellular and network level where they facilitate glutamate-mediated neurotransmission and acutely increase cortical excitability (36–39). Our results reveal that these acute alterations in the medial prefrontal cortex selectively attenuate negative affective biases during memory retrieval in our rat model. Furthermore, we found that the mechanism underlying this (phase 1, Figure S12) was independent of protein synthesis and so likely corresponded to early synaptic long-term potentiation/long-term depression (LTP/LTD) (40, 41). Differences among the three drugs emerged when looking at their post-acute effects, that is, 24 hours after dosing. We observed that low doses of either ketamine or psilocybin generated a state where negatively biased memories could be reactivated and re-learned with a more positive affective valence. In contrast, at 24 hours after dosing with scopolamine and at higher doses of ketamine there was only a sustained attenuation of negative bias. This suggests that the phase 2 mechanism may be dissociable from phase 1 and that engagement of other receptors or circuits by scopolamine and a higher dose of ketamine could prevent re-learning of the affective bias. Indeed, as the dose of ketamine was increased, the selectivity of effects was reduced, with doses of 10 and 25 mg/kg failing to generate the positive bias at 24 hours post dosing and 25 mg/kg inducing generalised impairments in memory retrieval. Our study confirmed that for ketamine, the phase 2 mechanism involved the medial prefrontal cortex and was dependent on protein synthesis, suggestive of a mechanism involving late LTP/LTD or structural neural plasticity (26, 42, 43). Although the positive bias was observed 24 hours post dose, anisomycin was delivered before ketamine in this experiment, and so initiation of the phase 2 mechanism must have occurred during the acute drug effects but was stable for at least 24 hours. This is further supported by our finding that the reward-associated digging substrate cue reactivated one hour post ketamine treatment could alter the affective bias observed at 24 hours indicating that experience-dependent neural plasticity may underlie phase 2. Further exploration into the downstream mechanisms mediating phase 1 and 2 effects are needed. Other studies with these RAADs suggest convergence on neural plasticity signaling pathways involving BDNF, mTOR and TrkB as well as other potential mediators (21, 42, 44). Induction of these downstream signaling molecules has also been linked to induction of neuronal spine and dendrite formation that may contribute to the sustained effects of RAADs (42, 45). One of the challenges in interpreting these findings has been the dependence on behavioral readouts such as the forced swim test that has limitations (46, 47).

Further differences among the three RAADs were that only low doses of psilocybin induced positive affective biases when administered prior to new experiences. Positive biases during new learning experiences have previously been associated with conventional antidepressants (12, 16) and may further contribute to the clinical benefits of psilocybin. The neural mechanisms mediating these effects have yet to be elucidated but studies with the mixed re-uptake inhibitor, venlafaxine, suggest they involve the amygdala (14). In the new learning protocol in our study, psilocybin’s effects were dose-dependent but with a negative affective bias observed with the highest dose tested. A similar trend was also seen in the effects on memory retrieval with the higher doses tested not exhibiting any additional beneficial effects and inducing greater variability in the data. This requires further investigation, but evidence from clinical studies and other behavioral readouts in rodents, suggest that doses of psilocybin greater than 0.3mg/kg induce greater psychedelic effects.

Our key finding is the 24 hour effect and that experience-dependent neural plasticity during the acute phase of RAAD treatment can have long lasting effects on biased memories that ultimately underpin mood. For animals to exhibit a positive affective bias 24 hours after RAAD treatment, the memory associated with the negative affective state manipulations must have been preferentially reactivated and re-learned relative to the memory learned under control conditions. This was confirmed in the cue reactivation test where, by re-activating the control memory and thus making it relatively more salient, we were able to attenuate the 24 hour effect. Our rodent data also suggest that the affective bias modification not only shifted from negative to neutral, but shifted to a relatively more positive bias. This would align with human imaging data where ketamine and psilocybin treatment have been suggested to disrupt networks thought to generate these negative biases such as the default mode network (48, 49).

Although the different RAADs tested in this study have all been shown to induce rapid and sustained antidepressant effects in clinical populations, the duration of the effects varies. Notably, clinical trial data is more limited for scopolamine and psilocybin than for ketamine although positive clinical findings have been reported for scopolamine and psilocybin including a recent phase 2 multi-center clinical trial with psilocybin (5). Further studies are required to determine whether temporal differences in efficacy among these RAADs are specifically related to affective bias modifications mediated by the drug or whether additional factors are involved. Based on the clinical data available, ketamine’s efficacy develops from approximately one hour post-infusion and lasts for 1-2 weeks in MDD patients (50), with the peak of amelioration of depressive symptoms occurring 24 hours after the infusion (2, 51). Using a block design and intermittent dosing, scopolamine induced an improvement in mood in patients with MDD within days and these effects were sustained for several weeks (4, 52). Psilocybin’s antidepressant effects have been observed from the first day of treatment and can last for at least 6 months (5, 53). In our affective bias test in rats, psilocybin showed beneficial effects on affective biases associated with past and future memories, whereas ketamine’s effects were limited to the modification of biased memories and scopolamine’s effects were limited to attenuation of a negative affective bias. Ketamine’s effects were also dose dependent and align with clinical data suggesting that higher doses of ketamine do not have antidepressant effects (23–25).

There are a number of limitations to our study. Our study only used male animals and our results may not be generalisable to female rats. We have run our affective bias test in female rats and also using different rat strains and have found consistent effects in terms of both positive and negative affective bias modification (15). In our meta-analyses of these data, our findings suggest sex differences would only represent a small effect and studies designed to identify sex differences would therefore require high sample sizes. We also only investigated the effects of RAADs in normal laboratory animals and using a within-subject study design. The affective bias test depends on an individual animal making choices based on their past experiences of two associative memories, with one memory learned during an affective state manipulation and the other learned under control conditions. We have also only investigated affective biases associated with reward-related learning and memory. Whether these effects can be generalised to other cognitive domains and aversive as well as appetitive memories requires further investigation. Future studies are needed to integrate the affective bias test and rodent disease models of MDD.

In summary, affective bias modification by RAADs may represent a neuropsychological mechanism that could explain the sustained improvements in mood that arise and persist after a single RAAD dose. Our affective bias test and rat model will be useful to explore the underlying biological and experience-dependent factors that contribute to these effects. Our study results also support a specific biological mechanism underlying the rapid and sustained antidepressant effects of RAADs and suggest that their clinical benefits are more than an exaggerated placebo response arising from their powerful dissociative and psychedelic effects (54).

## Materials and Methods

### Study design

We designed this study to test the hypothesis that the rapid antidepressant effects of drugs such as ketamine, psilocybin and scopolamine may be related to their ability to modulate affective biases. Specifically, we predicted that low, clinically relevant doses of ketamine, psilocybin and scopolamine would attenuate a negative affective bias associated with a reward memory in our affective bias test in rats. We predicted these effects would be specific and no effects would be observed when the same doses were tested in a control memory test. We also tested whether the effects of these RAADs were sustained. We explored the re-learning effect of ketamine further by infusing ketamine into the medial prefrontal cortex of male rats and used a protein synthesis inhibitor anisomycin and a cue reactivation test to explore contributions of neural plasticity and experience-dependent factors in the effects observed.

We tested male rats using our affective bias test and reward learning assay in combination with systemic administration of ketamine, psilocybin or scopolamine, or targeted brain infusions of ketamine only. We focused the brain infusion studies on the medial prefrontal cortex using animals implanted with intracerebral cannulae to facilitate local administration of either ketamine or the protein synthesis inhibitor.

All experiments used a within-subject design where each animal received all treatments using a fully randomised study design. With the exception of the cue reactivation study, researchers were blinded to treatment throughout the experiment and analysis. Blinding was only broken after all inclusion and exclusion criteria had been applied and the statistical analysis completed. For the memory retrieval experiments, animals that did not exhibit the expected negative bias under vehicle treatment were excluded. This led to the removal of one animal from the acute retrieval study with psilocybin treatment (0.1-0.3mg/kg), three animals from the acute retrieval study with ketamine-anisomycin infusion and one animal from the 24-hour retrieval study with ketamine infusion. We also excluded one outlier (more than 2 standard deviations from the group mean) from the ketamine new learning study. Animals that completed less than 15 trials during the choice test were also excluded from the choice bias analysis. This included three animals from the reward learning assay with ketamine (25mg/kg) treatment, and one animal from the ketamine (1.0-25.0mg/kg) 24 hour retrieval study. All animal experiments were conducted according to the UK Animals (Scientific Procedures) Act 1986 and under a project license from the UK Home Office. All experimental procedures were approved by the University of Bristol Animal Welfare and Ethical Review Body.

### Animals

Ten separate cohorts of male Lister Hooded rats (Envigo, UK) were used in these experiments (n=11-16 per group; Table S9). This study only used male rats, however, previous studies suggest similar affective biases are observed in both sexes (15). Animals were pair-housed in standard enriched laboratory cages under a 12:12 hour reverse light-dark cycle (lights off at 08:00h) and in temperature-controlled conditions (21±1°C). Rats were food restricted to approximately 90% of their free-feeding weights matched to the normal growth curve [~18 g of food per rat/day laboratory chow (Purina, UK)] and were provided with water ad libitum. The behavioral procedures and testing were performed during the animals’ active phase between 09:00h and 17:00h.

### Affective Bias Test

#### General protocol

##### Training

The apparatus and detailed training protocol followed that of Stuart et al. (16). Animals were first trained to dig in ceramic bowls containing sawdust over 5 days with increasing levels of difficulty until the final session when they completed a novel discrimination test to confirm they had learnt the task rule, which was that digging in the correct substrate led to finding a food reward (Movie S1). Choice of the reward-paired substrate was marked as a ‘correct’ trial, digging in the unrewarded substrate was classified as an ‘incorrect’ trial and if an animal failed to approach and explore the bowls within 30 seconds, the trial was recorded to be an ‘omission’. Trials were continued until the rat achieved six consecutive correct choices for the reward-paired substrate. The discrimination session allowed us to confirm that the animals could achieve our learning criterion of six consecutive correct trials in less than 20 trials. Once animals successfully reached criteria in the discrimination session, they were considered trained. All animals then progressed to a reward learning assay protocol to confirm that they would exhibit a reward-induced bias and were therefore performing the task correctly and making their choice based on the memory associated with the digging substrate.

##### Testing

Each week was composed of four pairing sessions (one per day) to generate two independent cue-specific memories. Using a within-subject design, each animal learnt a specific substrate-reward association under either a control or affective state-induced condition followed by a choice test on the fifth or sixth day of the same week, where retrieval of the memories was tested with or without drug pre-treatment. Details of the pairing session and choice test procedures are given in the supplementary methods and a list of substrates used is included (Tables S10, S11). All drug treatments, pairing substrates and order of presentation were fully randomised in all studies.

Affective biases generated by this protocol were quantified during the choice test when the two previously rewarded substrates (‘A’ and ‘B’) were presented at the same time over 30 spatially randomised trials. In order to keep rats motivated a single 45mg food pellet was placed in either bowl using a random schedule with a probability of one in three, so that rats randomly received a reward (i.e. substrate ‘A‘ contained a pellet on 10 of the 30 trials, as did substrate ‘B’; on no trials were both bowls baited). Both bowls also had a pellet crushed and placed in the substrate to reduce the likelihood of the animal using odor to find the reward. The animals’ choices and latency to dig were recorded.

Putative antidepressant effects of RAAD treatment were tested in one of three versions of the affective bias test, which enabled dissociation of different neuropsychological mechanisms and effects on new learning versus acute or sustained effects on previously learnt, biased memories. To investigate the putative effects of RAADs on past experiences, we first replicated the acute protocol we had previously used for our ketamine study (14) where the drugs were administered 1 hour before the choice test. To explore the sustained effects of RAADs, we tested animals in the choice test 24 hours after drug treatment with the test drug administered 24 hours after the last pairing session and 24 hours before the preference test. To test the effects on new experiences drug treatments were administered before learning (days 1-4), and were counterbalanced with a vehicle control.

In the cue reactivation study, male rats underwent a six-day modified study design (Figure 4, Table S11C). From days 1 to 4 each animal learnt specific substrate-reward associations under either a vehicle-induced condition or FG7142-induced condition followed by a cue reactivation protocol on the fifth day and a choice test on the sixth day of the same week. On day 5, animals were treated with either vehicle or ketamine 1.0mg/kg followed by a cue reactivation protocol one hour later. The cue reactivation protocol involved placing the animal either in an empty affective bias test arena (vehicle no cue reactivation or ketamine no cue reactivation) or in the affective bias test arena with a bowl containing the FG7142-paired substrate (ketamine + re-exposure to FG7142 cue) or vehicle-paired substrate (ketamine + re-exposure to vehicle cue) for 3 seconds. Animals were tested 24 hours after the drug treatment and cue reactivation protocol. Due to the experimental design of this study the experimenter was not fully blinded to treatment.

### Reward learning assay

The reward learning assay was used to establish the specificity of the treatments in relation to affective state-induced biases as opposed to a general impact on memory. The reward learning assay used a similar protocol to the affective bias test, with 4 pairing sessions and a choice test except that animals remained in the same affective state throughout the one-week protocol and learnt to associate the one reward-paired digging substrate with a high (2 pellet) and the other with a low (1 pellet) reward. The effects of RAAD treatment on retrieval of these memories and the reward-induced bias were tested by administering the drug one hour before the choice test to check for any non-specific and acute effects on memory (Table S11B).

### Drugs

The drugs used to induce a negative affective bias in rats were corticosterone (10mg/kg, s.c.) and FG7142 (3mg/kg, s.c.). The RAADs tested were ketamine (1, 10, 25mg/kg), scopolamine (0.1mg/kg), psilocybin (COMP360, an investigational medicinal drug/product that does not have Marketing Authorisation and is not approved for therapeutic use, other than in a clinical trial environment) (0.1, 0.3, 1.0mg/kg, i.p.), venlafaxine (3mg.kg, i.p.) and anisomycin (100 ug/ul, 1ul infusion) (Table S9).

The doses for corticosterone, FG7142 and ketamine were based on previous studies (13–15, 40). Anisomycin (44) and scopolamine doses were chosen based on our judgement bias task dose response studies (35) and psilocybin doses were based on a previous head-twitch response study (55). For corticosterone or FG7142-induced negative affective biases, we selected a sub-maximal dose previously shown to induce a robust negative affective bias in the affective bias test (15, 16, 22).

Ketamine doses were considered based on available pharmacokinetic data for humans and rodents (56, 57), as well as by calculating the animal equivalent dose (58). The ip route of administration provided rapid drug absorption and distribution and avoided the need for restraint or a surgical intervention as required for intravenous (iv) infusions. We used a 1mg/kg ip dose of ketamine to provide a similar dose and plasma concentration to that achieved with an iv dose in humans; 10 and 25mg/kg doses given ip in rats were considered equivalent to high doses in humans.

Intraperitoneal injection procedures were done using a low-stress, non-restraint method developed in our research group (59). All animals were habituated to their holding position required for ip dosing for five days prior to the experiments. All subcutaneous injections were performed with minimal animal restraint and injected on their left or right flank (changing daily).

In all experiments, a within-subject design was used, with the experimenter blinded to treatment and with a fully counterbalanced experimental design. In studies testing psilocybin, the number of head twitches and wet dog shakes were scored (Tables S5-S8).

### Medial prefrontal cortex cannulation and infusion

For experiments involving infusion of ketamine or anisomycin into the medial prefrontal cortex, male rats from cohorts 2 and 8 were first implanted with a bilateral guide cannula (32-gauge, Plastics One, UK) into the medial prefrontal cortex (stereotactic coordinates from Bregma [+2.70mm anterior/posterior (AP), ±0.80mm medial/lateral (ML) and −2.1mm dorsal/ventral (DV) from dura] (45).

After the recovery period, all animals were habituated to the infusion procedure during two sessions on separate days. During experimental infusions, each rat was lightly restrained while the dummy cannula was removed, the injector was placed through the guide cannula for a 1 minute pre-infusion, 2 minutes for infusion of vehicle or drug (1ug/ul ketamine, 1.0 μl per site, with a flow rate of 0.5 μl per minute) and for 2 minutes post-infusion to allow diffusion of the vehicle/drug into the surrounding tissue. All animals were infused with anisomycin (100ug/ul) or vehicle (PBS) 30 minutes prior to ketamine injection (systemic 1mg/kg, i.p.) or vehicle injection (fully counterbalanced design). Animals were then tested, either one hour post treatment for the acute modulation of a negative affective bias or 24 hours post treatment for sustained modulation of a negative affective bias. At the end of the study, cannulated rats were killed by transcardiac perfusion with PBS followed by 4% paraformaldehyde under terminal sodium pentobarbitone anaesthesia and the brain was removed, sectioned and stained with cresyl violet to determine cannula position. All animals were included in the post-histological verification analysis.

### Quantification of head twitch and wet dog shakes induced by psilocybin

The head-twitch response is defined as a rapid side-to-side head movement, whereas the wet-dog shakes included a head-twitch response and a whole-body shake. Psilocybin was administered 60 minutes prior to a substrate-reward pairing session or choice test. Animals were observed for 10 minutes during the pairing session or 15 minutes during the choice test, and the total number of head-twitch responses and wet dog shakes were scored (Tables S5-S8).

### Data analysis

Data were analysed using SPSS Statistics 28 and figures were created using GraphPad Prism 9.4.0 (GraphPad Software, USA). Choice bias score was calculated as the number of choices made for the drug-paired substrate (affective bias test) or two pellets-paired substrate (reward learning assay) divided by the total number of trials multiplied by 100 to give a percentage. A value of 50 was then subtracted to give a score where a choice bias towards the drug-paired substrate gave a positive value and a bias towards the control-paired substrate gave a negative value. For the memory retrieval studies involving a FG7142 or corticosterone-induced negative bias, animals that did not exhibit the expected negative bias under vehicle treatment were excluded. Values that were more than 2 standard deviations from the group mean were also excluded. Data from animals that completed less than 15 trials during the choice test were removed from the choice bias analysis. Choice bias scores and response latency scores during the choice test were analysed using a repeated measures ANOVA with treatment as the within-subject factor, and as a post-hoc analysis pairwise comparisons were made using a two-tailed paired t-test or Dunnett’s test depending on the number of group comparisons. Individual positive or negative affective biases were also analysed using a one-sample t-test against a null hypothesis mean of 0% choice bias. For each animal, mean trials to criterion and latency to dig during the affective bias test pairing sessions and choice test were analysed using a repeated measures ANOVA with treatment as the factor or a two-tailed paired t-test, with post-hoc pairwise comparisons made using a two-tailed paired t-test (new learning studies) or two-tailed paired t-test comparison between control (vehicle/low reward:1 pellet) and treatment/manipulation (corticosterone/FG7142/high reward: 2 pellets) for each week (drug-induced negative bias retrieval studies and reward learning assay). Analysis of the choice latency and trials to criterion was made to determine the presence of any nonspecific effects of treatment, such as sedation. A Shapiro-Wilk test was used to determine a normal distribution for the % choice bias, trials to criterion, and mean latency to dig during pairing sessions and choice test. Mauchly’s sphericity test was used to validate a repeated measures ANOVA. Effect sizes are presented as Cohen’s d for t-tests and post-hoc tests, or η2 for ANOVA. Data for the number of head twitches and wet dog shakes were analysed using non-parametric methods, the Kruskal Wallis test followed by Mann Whitney test post-hoc pairwise comparisons.

## Supplementary Material

Supplementary Material

## Data Availability

All data are available in the main text or the supplementary materials. Individual-level data for all studies are available on the Open Science Framework at https://doi.org/10.17605/OSF.IO/625SF. Psilocybin (COMP360) was provided by COMPASS Pathways to the University of Bristol under a materials transfer agreement.
