# Supplementary material for: Rapid-acting antidepressant drugs modulate affective bias in rats
Source: Sci Transl Med. Author manuscript; Available in PMC 2024 Jan 25. (PMC7615567; doi:10.1126/scitranslmed.adi2403)
Supplement: Supplementary Material [file EMS193444-supplement-Supplementary_Material.pdf]

Supplementary materials

Additional information for materials and methods

Animals and housing

All rats weighed between 300-350g at the start of training (12-13 Weeks of age). The sample size was based on our previous affective bias test (ABT) studies and a meta-analysis which suggested a medium to large effect size for the drug-induced negative bias and reward-induced bias in Lister Hooded rats (13,14). All animals were pair-housed in standard enriched laboratory cages (55x35x21cm) with woodchip, paper bedding, cotton rope, wood chew, cardboard tube and red Perspex house (30x17x10cm), under a 12:12h reverse light-dark cycle (lights off at 08:00h) and in temperature-controlled conditions (21±1°C). The behavioural procedures and testing were performed during the animals' active phase between 09:00h and 17:00h.

Affective Bias Test (ABT)

General protocol

Training: The ABT testing was carried out in a Perspex® arena (40x40cm) with two ceramic bowls (Ø 10cm) and a trio of digging substrates (reward-paired substrates - 'A' or 'B' versus unrewarded substrate - 'C', matched for digging effort and counterbalanced across subjects; for details, see Supplementary Table S1). Prior to ABT training animals underwent a five day habituation to handling with positive reinforcement (reward pellets) and two habituation sessions to the ABT arena (first without bowls, substrate or reward and second with empty bowls); rats were individually placed into the arena and allowed to explore for 10min. Further training consisted of three digging training sessions (20 trials per session) with a bowl filled with increasing amounts of digging substrate (sawdust) and a food reward (45mg purified rodent tablets, Test Diet, Sandown Scientific, UK). On the first day of digging training, each rat was placed in the arena and given 30s to approach and explore the empty bowl (without substrate) containing two pellets per trial. When the pellets were found and consumed, the trial was completed, and the rat was removed from the arena and the pellets were replenished in the bowl. During the next digging training session, each rat was given 30s to explore the bowl and start digging for a single pellet buried within 1 cm of sawdust. Following 20 trials in which the pellet was found and eaten, each rat was moved onto the final training session in which a single pellet was buried within 2 cm of sawdust. Once each animal was able to find a pellet within 30s on 10 consecutive trials (within a maximum 20 trials), the digging training was complete.

Following the training sessions, animals underwent a discrimination session allowing them to explore two bowls with two novel digging substrates (reward-paired substrate with single pellet versus unrewarded substrate). On each trial, the animal was individually placed in front of the two bowls. Once the animal made a choice by starting to dig in one bowl, the other bowl was removed by the experimenter. An example of a single discrimination trial is illustrated in supplementary movie S1. Choice of the reward-paired substrate was marked as a 'correct' trial, digging in the unrewarded substrate was classified as an 'incorrect' trial and if an animal failed to approach and explore the bowls within 30s, the trial was recorded as an 'omission'. Trials were continued until the rat achieved six consecutive correct choices for the reward-paired substrate. The discrimination session allowed us to confirm that the animals could achieve our learning criterion of six consecutive correct trials in less than 20 trials. Once animals successfully reached criteria in the discrimination session, they were considered trained and progressed to testing in the reward learning assay. As detailed below, this test was carried out over 5 days and used to check that the cohort was correctly performing the task and at population level reward-induced positive bias was observed before animals progressed to studies involving affective state-induced biases and their modulation by RAADs.

Testing: Each week was composed of four pairing sessions (one per day) to generate two independent cue-specific memories (Supplemental figure S1). During the pairing sessions, each trial involved presenting the rat with a choice between two bowls containing two different digging substrates, one of which was reward-paired (substrate 'A' or 'B', counter-balanced across subjects and manipulation) and contained a single 45mg reward pellet, and the other of which was unrewarded (substrate 'C'). Substrate C was kept the same for all four pairing sessions and a reward pellet was crushed into the bowl and mixed within the substrate, to prevent choices based on odour. One of substrates 'A' or 'B' was presented during pairing sessions on days 1 and 3, and the other was presented on days 2 and 4, with order counterbalanced across subjects (see Supplemental tables S2A-C).

Drugs

Drugs tested in the new learning studies and reward learning assays were ketamine, psilocybin and scopolamine (for details, see Supplementary Table S1). Corticosterone (10.0 mg/kg, administered subcutaneously, with pre-treatment time 30 min. prior to the individual substrate-reward pairing sessions (t=−30 min.)) and FG7142 (3.0 mg/kg, administered subcutaneously, t=−30 min.) were purchased from Sigma-Aldrich, UK. For systemic studies, scopolamine (0.03, 0.1 mg/kg, administered intraperitoneally, t=-60 min. and t=-24hrs) was purchased from Tocris, UK; psilocybin (0.1, 0.3, 1.0mg/kg, administered intraperitoneally, t=-60 min. and t=-24hrs) was supplied by COMPASS Pathways; venlafaxine (3.0mg/kg, administered intraperitoneally, t=-60 min.) was purchased from LKT Laboratories, Inc. For systemic and infusion studies, ketamine (1.0 mg/kg and 1µg/µl, administered intraperitoneally and by infusion, respectively, t=-60 min. and t=-24 hrs,) was purchased from Sigma-Aldrich, UK and anisomycin (100 ug/ul, administered by infusion, t=-90min. and t=-24.5 hrs) from Tocris, UK. Drugs were dissolved in vehicle solutions as follows: for corticosterone, it was 5% DMSO (VWR Chemicals, UK) and 95% sesame oil (Sigma-Aldrich, UK), for FG7142 it was 5% DMSO, 10% cremophor and 85% sterile saline, and for scopolamine, psilocybin, venlafaxine and ketamine it was 0.9% sterile saline. For mPFC infusions ketamine was dissolved in PBS and anisomycin in HCl/PBS and its final pH was established at 7.4. All drugs were freshly prepared every day and they were administered in a dose volume of 1.0 ml/kg and 1.0 ul per site, in systemic and infusion studies, respectively.

Medial prefrontal cortex cannulation procedure

The surgical procedures were performed under inhalation anaesthesia of the isoflurane/O2 mix. The cannula was fixed to the skull with gentamicin bone cement (DePuy CMW, Johnson & Johnson, UK) and three stainless-steel screws. To reduce risks of infection and any blockage inside pins of the guide cannulae, dummy cannulae (Plastics One, UK) were placed inside and metal head caps secured on top. Animals received local anaesthetic during the surgery and following surgery were housed individually for ~3h and then allowed to fully recover for 11-14 days in pairs with free access to food and water. Postoperatively all animals were pair-housed in Techniplast high top cages (40.5 x 37.5 x 31 cm) with woodchip, paper bedding, cardboard tubes, wood chew and red Perspex houses (30 x 17 x 10 cm).

Infusion Procedure

The first habituation session involved animals being gently restrained, the dummy cannula removed, cleaned and then placed back. The second session involved dummy removal followed by insertion of the bilateral injection cannula (injector, 33-gauge, Plastics One, UK) extending 2.5mm beyond the length of the guide cannula into the mPFC and left in position for 5 min. without infusion. The injector was then removed, and the dummy cannula and head cap replaced.

Data analysis

For the memory retrieval studies involving a FG7142 or corticosterone-induced negative bias, animals which did not exhibit the expected negative bias under vehicle treatment were excluded. Applying this exclusion criteria led to the removal of four animals, one from the

retrieval at 1h study with psilocybin (0.1-0.3mg/kg) treatment, three from the retrieval at 1h study with ketamine-anisomycin infusion and one from the retrieval at 24hrs study with ketamine infusion. We have also excluded one outlier (more than 2SD) from the ketamine new learning study, and animals that completed less than 15 trials during choice test, three animals from the reward learning assay with ketamine (25mg/kg) treatment, and one animal from the ketamine (1.0-25.0mg/kg) retrieval at 24hrs study. Their data was removed for all treatments in that study for choice bias data.

Supplementary Figures

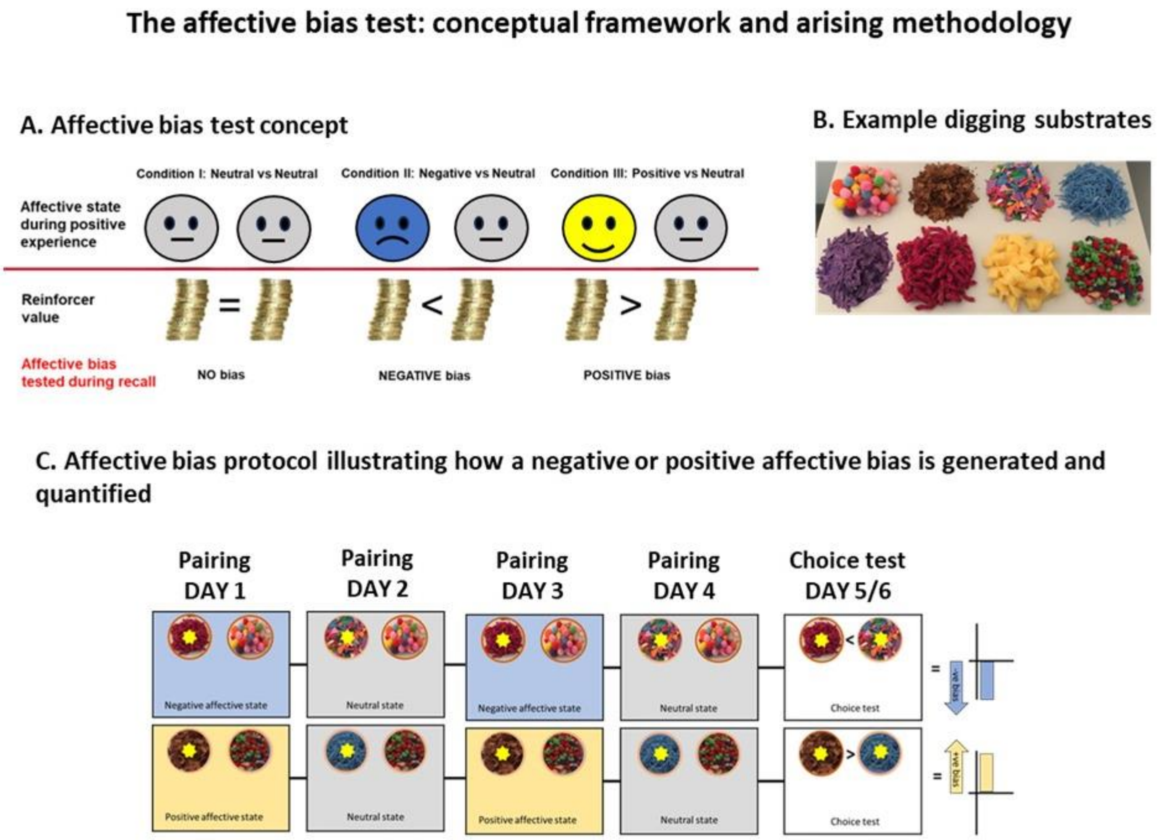

Figure S1: The conceptual framework for the affective bias test (ABT) is outlined in panel A. The task builds from the observation that patient with major depressive disorder attribute less value to the memory of positive experiences indicative of a negative affective bias in learning and memory. The ABT was designed to enable the direct quantification of these affective biases in non-human species by generating two independent memories learnt under either an affective state manipulation or neutral/control state. Based on the concept that the arising affective bias will change the relative value of the reinforcer; this can be directly quantified by measuring the relative preference for the experience learnt during the affective state manipulation versus the neutral state. This concept was translated into a task which uses associative learning where animals learn to associate a specific digging substrate (examples in panel B) with a food reward. The value of each, independent learnt experience, is kept the same with each animal undergoing four paring sessions over 4 days followed by a choice test as illustrated in panel C. During the choice test, the two previously rewarded substrates are presented together for the first time and the animals preference quantified over 30 randomly reinforced trials. By administering an affective state manipulation or test treatment prior to one of the substrate-reward pairing sessions the affective bias generated during learning can be quantified at retrieval using a choice test. Extensive pharmacological and psychosocial manipulations confirmed the predictions illustrated in panel C. The ability of a treatment to induce an affective bias is tested by administering the test substance before the pairing sessions. The ability of a treatment to attenuate a negative affective bias already generated is tested by first inducing a negative affective bias using an established negative state induction method e.g. acute corticosterone or the benzodiazepine inverse agonist, FG7142, the administering the treatment either acutely or 24hrs before the choice test.

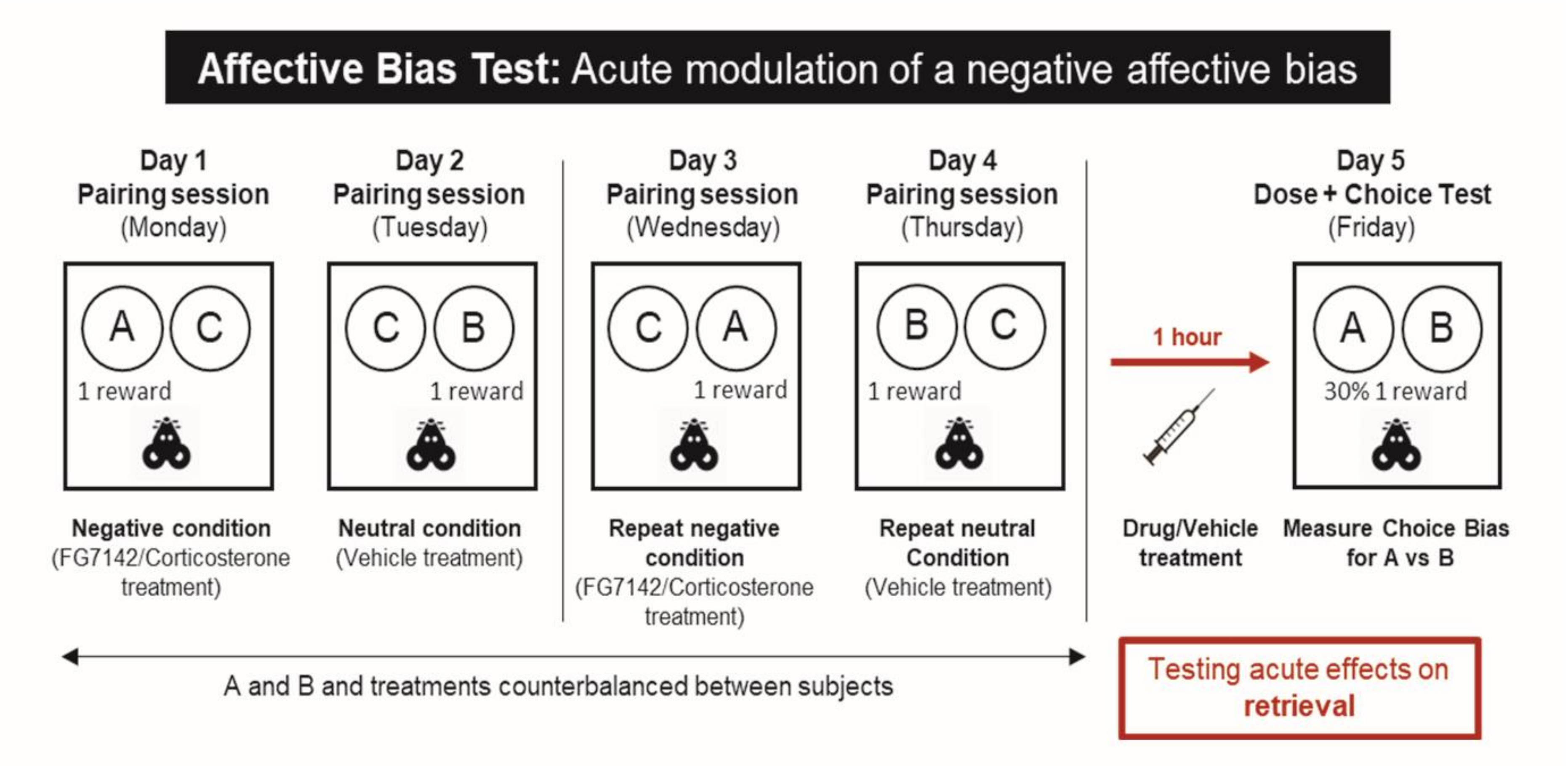

Figure S2: Overview of the affective bias test protocol used to investigate the acute effects of RAADs on a negative affective bias. Animals were treated with either FG7142 (3mg/kg) or corticosterone (10mg/kg) to induce a negatively biased memory. Affective biases were quantified using a choice test with the RAAD or vehicle administered 1 hour before testing to investigate the acute effects on retrieval.

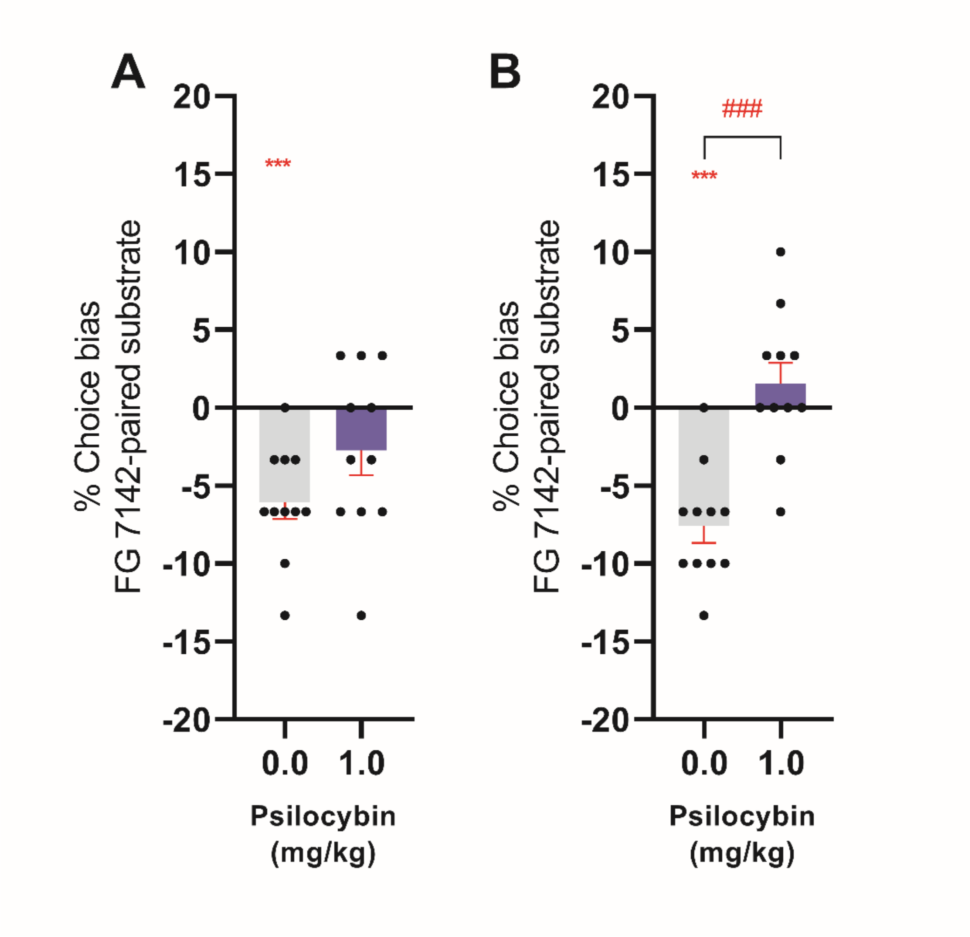

Figure S3: Effects of high dose psilocybin (1.0mg/kg) on acute (panel A) and sustained (panel B) modulation of a negative affective biases. To reduce the potential for carry over effects from the high dose of psilocybin and based on the data from the new learning protocol where an aversive effects was seen with 1mg/kg, the high dose was tested after the initial dose-response with a vehicle control group and fully counter-balanced. Although 1hr post treatment the negative bias was no longer evident in the one sample t-test, there was no significant difference between the vehicle and psilocybin group (panel A). The effects of 1mg/kg at 24hrs were also different from what was seen at the lower doses and although there was an attenuation of the negative bias, the inversion to a positive bias was not observed. Data shown as mean % choice bias ± SEM (bars) and individual data points (symbols), one sample t-test against a null hypothesised mean of 0% choice bias (***p<0.001) and pairwise comparisons using paired t-test (###p<0.001).

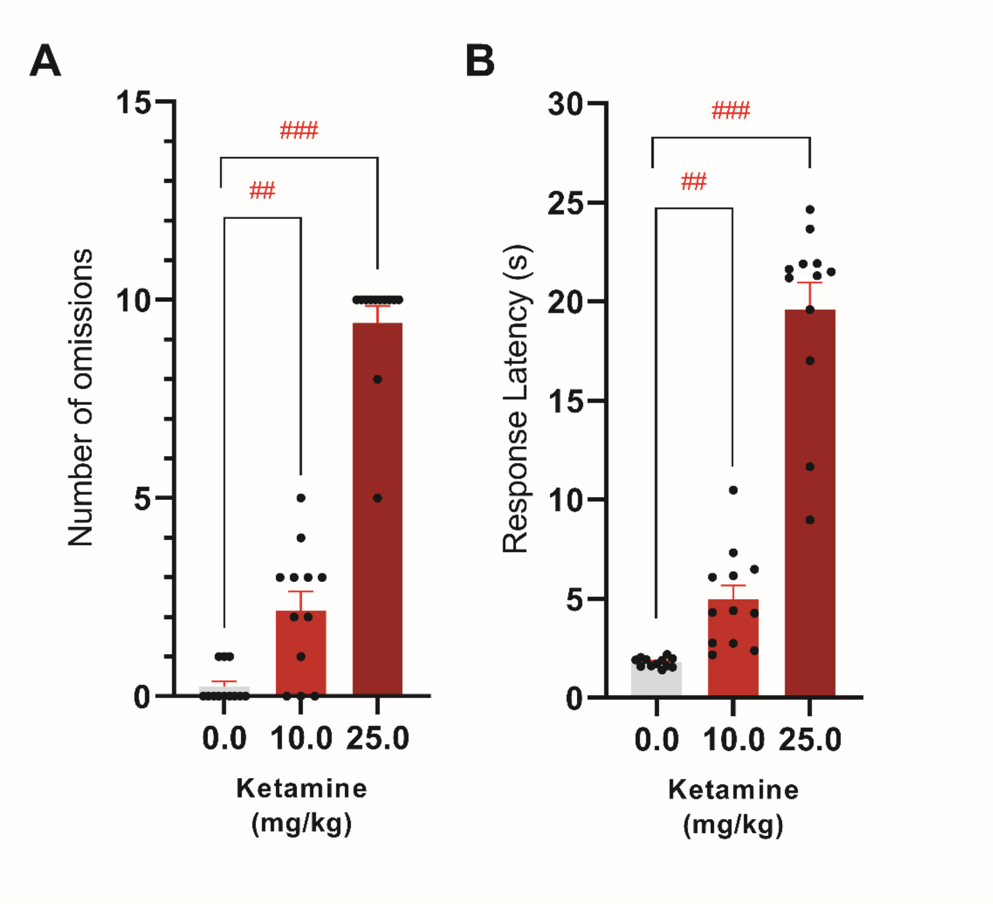

Figure S4: Effects of mid (10mg/kg) and high (25mg/kg) doses of ketamine on omissions (panel A) and latency (panel B) during the choice test. The choice test was terminated if animals had 10 or more omissions and although the choice data for the 25mg/kg dose is included for all subjects, only 2 animals completed the full 30 trials. Data shown as mean % choice bias ± SEM (bars) and individual data points (symbols), pairwise comparisons using paired t-test ($^{\#\#}p<0.01$, $^{\#\#\#}p<0.001$).

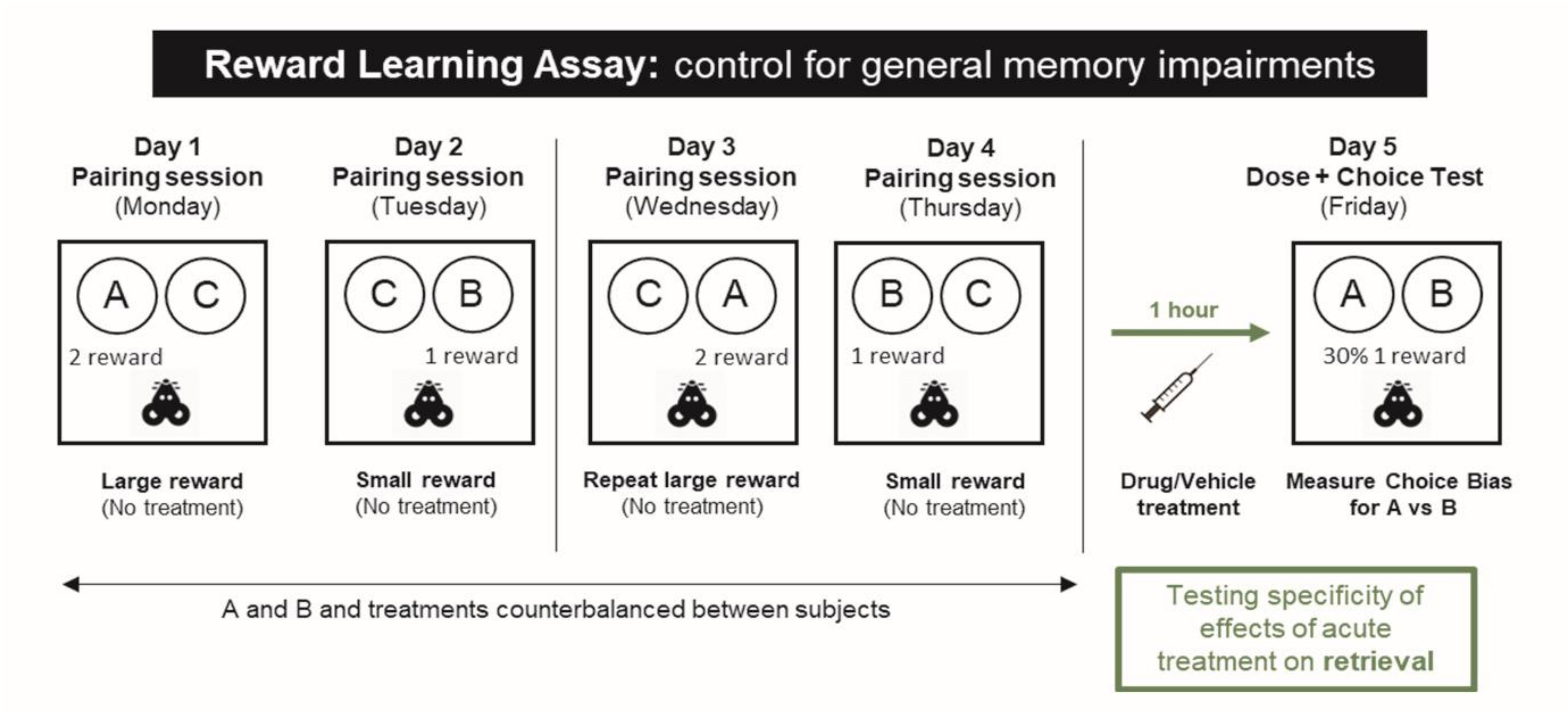

Figure S5: Overview of the reward learning assay protocol used to investigate the acute effects of RAADs on a reward-induced bias. Animals were in the same affective state throughout training and testing but learnt to associate one of the substrate-reward cues with a higher value reward (2 reward pellets) or a low value reward (1 reward pellet). The reward -induced bias was quantified using a choice test with the RAAD or vehicle administered 1 hour before testing to investigate the acute effects on retrieval.

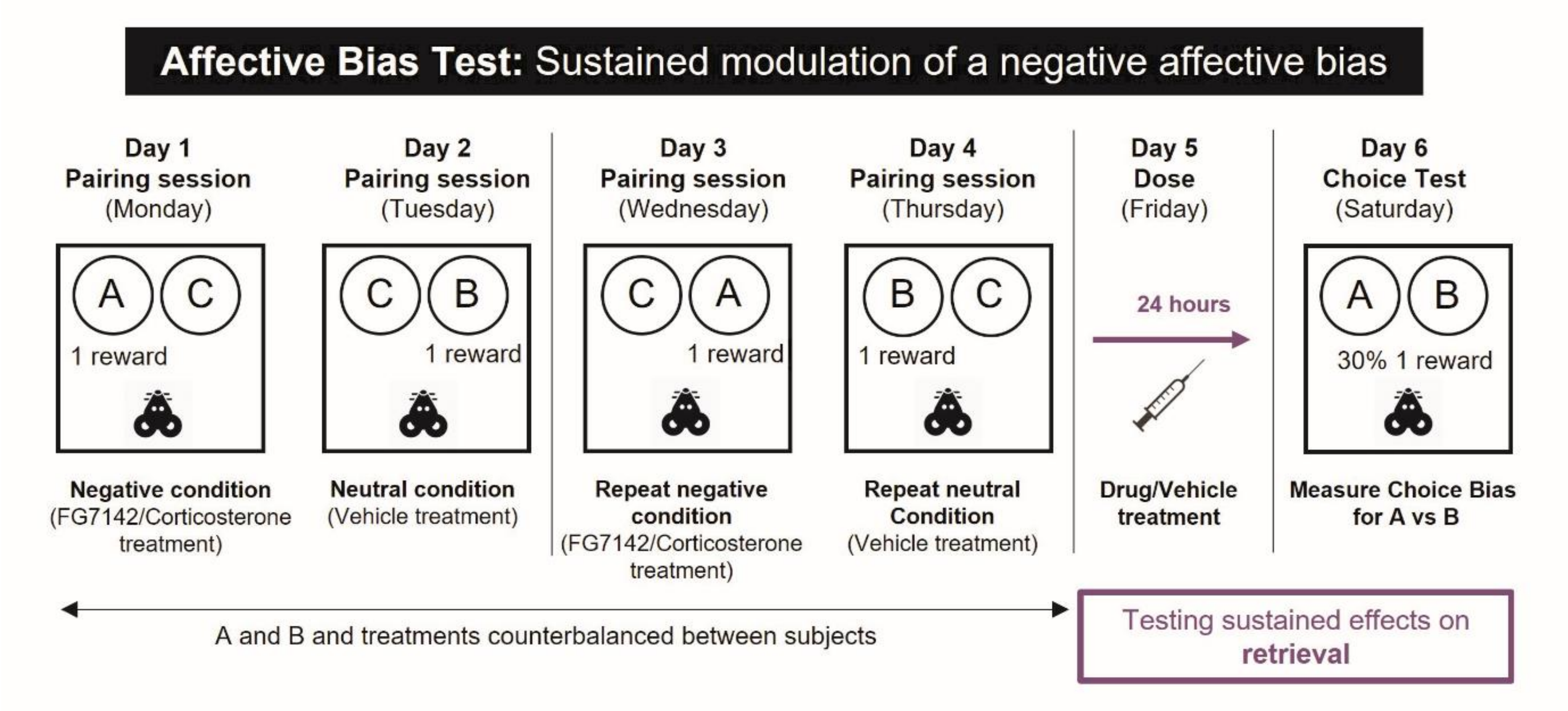

Figure S6: Overview of the affective bias test protocol used to investigate the sustained effects of RAADs on a negative affective bias. Animals were treated with either FG7142 (3mg/kg) or corticosterone (10mg/kg) to induce a negatively biased memory. Affective biases were quantified using a choice test with the RAAD or vehicle administered 24 hours before testing to investigate the acute effects on retrieval.

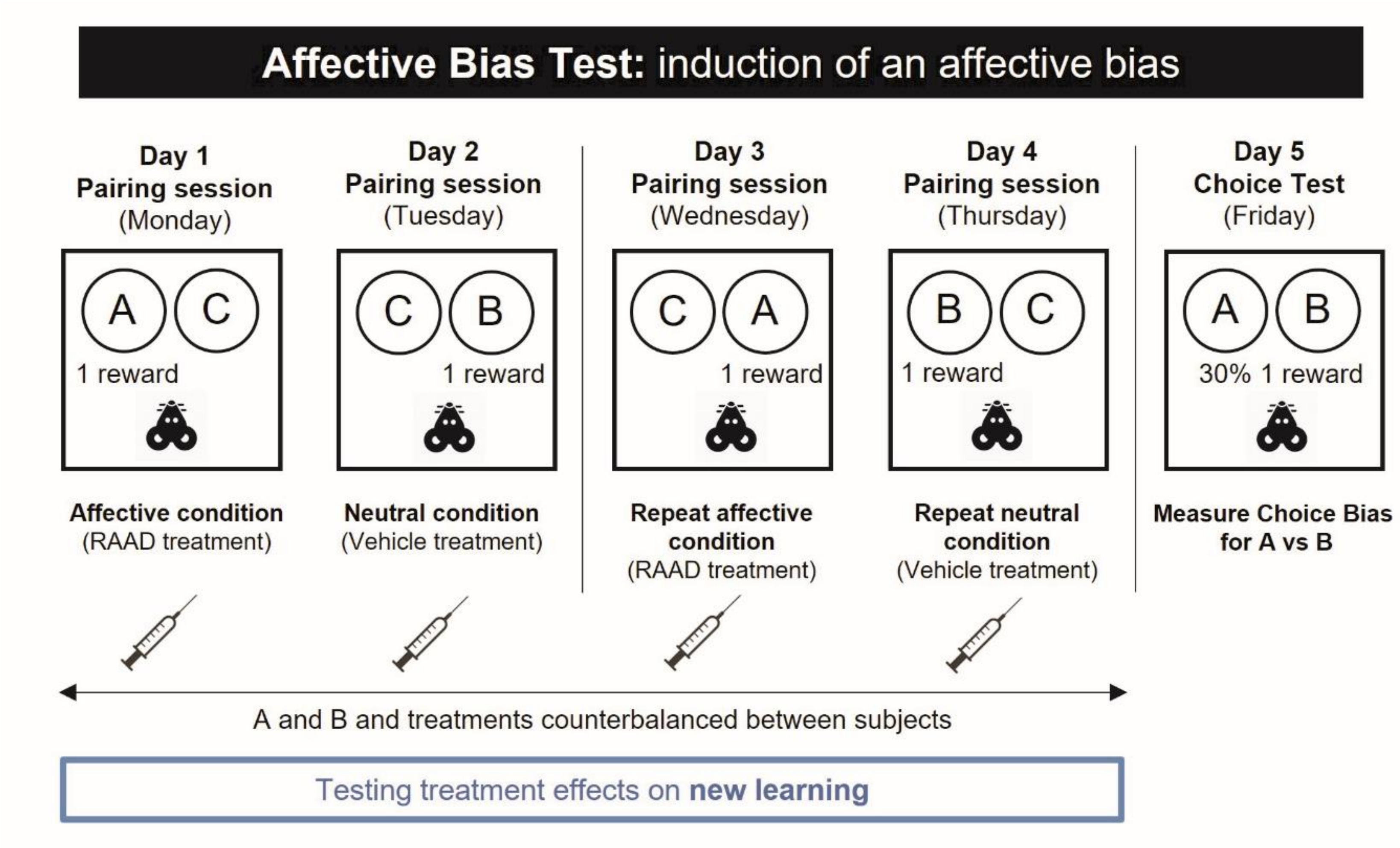

Figure S7: Overview of the affective bias test protocol used to investigate the effects of RAADs on new learning. Animals were treated with either the RAAD or vehicle prior to each of the independent substrate-reward association learning sessions with any arising affective bias quantified using a choice test 24 hours after the last pairing session.

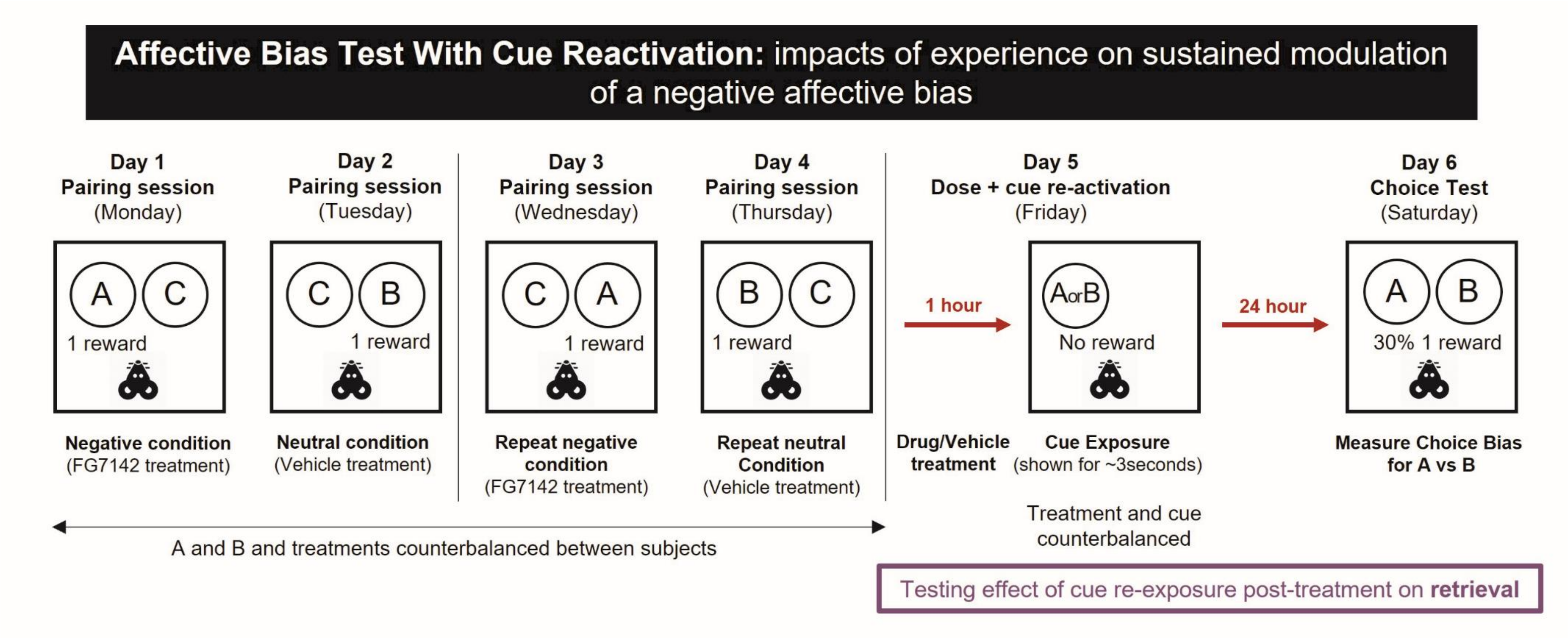

Figure S8: Overview of the affective bias test protocol used for the cue reactivation study. The protocol was similar to the method used to investigate the sustained effects of RAADs on a negative affective bias but included a cue-reactivation step on day 5, 1 hour after administration of ketamine (1mg/kg). Animals were treated with either FG7142 (3mg/kg) or corticosterone (10mg/kg) to induce a negatively biased memory and then re-exposed to either the FG7142-paired cue or the vehicle paired cue for 3 secs before being returned to their home cage. Affective biases were quantified using a choice test 24 hours after ketamine and cue-reactivation.

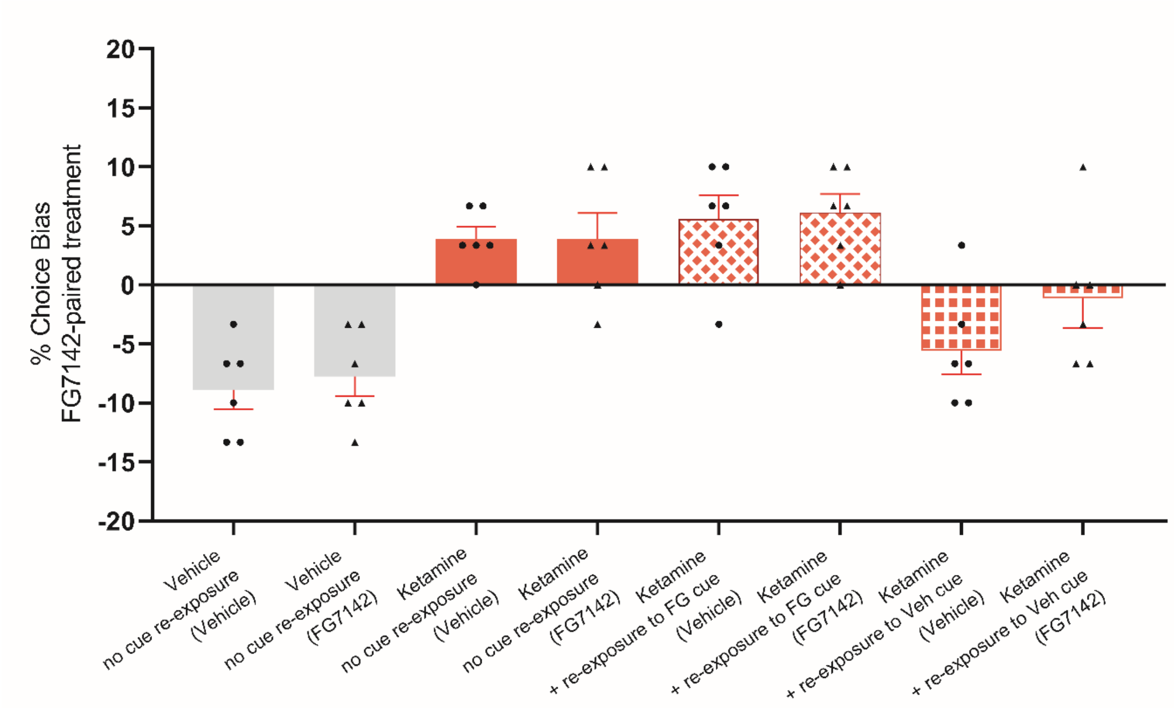

Figure S9: No evidence of a recency effect of the last pairing session during the choice test of the cue-reactivation study. In order to check whether the treatment-substrate-reward association learnt during the last pairing session had any impact on the memory retrieval in the cue re-activation study, we re-analysed the data based on whether the animal's last pairing session was the vehicle (Veh) or FG7142 (FG) treatment. Although the sample size for this analysis was reduced due to the counter-balanced design, there was no evidence that the last substrate-reward pairing session had any effects on the bias observed. The only group where there was a numerical difference observed was the ketamine (Ket) control re-exposure, mainly driven by a single value. Data shown as mean % choice bias ± SEM (bars) and individual data points (symbols, N=12 per treatment and 6 per condition), one sample t-test against a null hypothesised mean of 0% choice bias (*p<0.05, **p<0.01) and paired t-test with value adjusted for the number of comparisons.

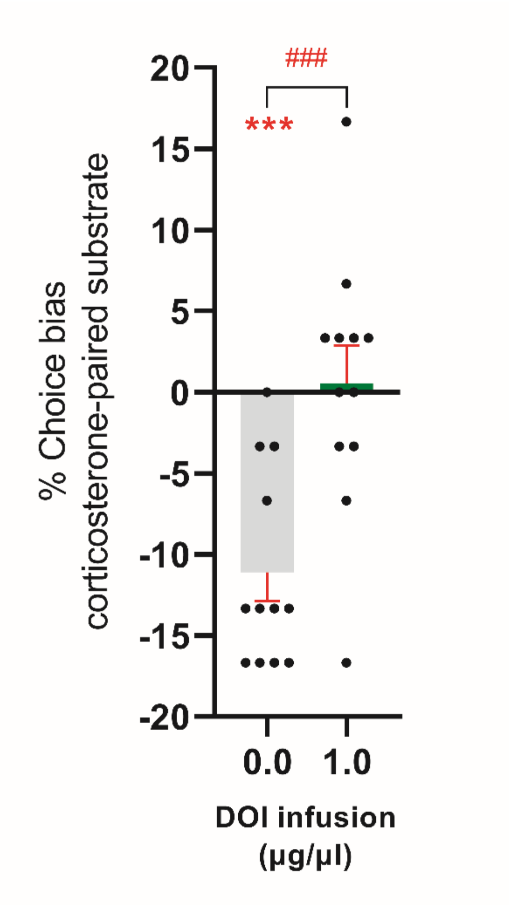

Figure S10: Infusion of the 5-HT$_{2A}$ agonist, DOI (1ug/ul), into medial prefrontal cortex induced similar acute effects to those seen with ketamine. Data shown as mean % choice bias ± SEM (bars) and individual data points (symbols, N=12), one sample t-test against a null hypothesised mean of 0% choice bias (***p<0.001) and paired t-test ($^{###}$p<0.001).

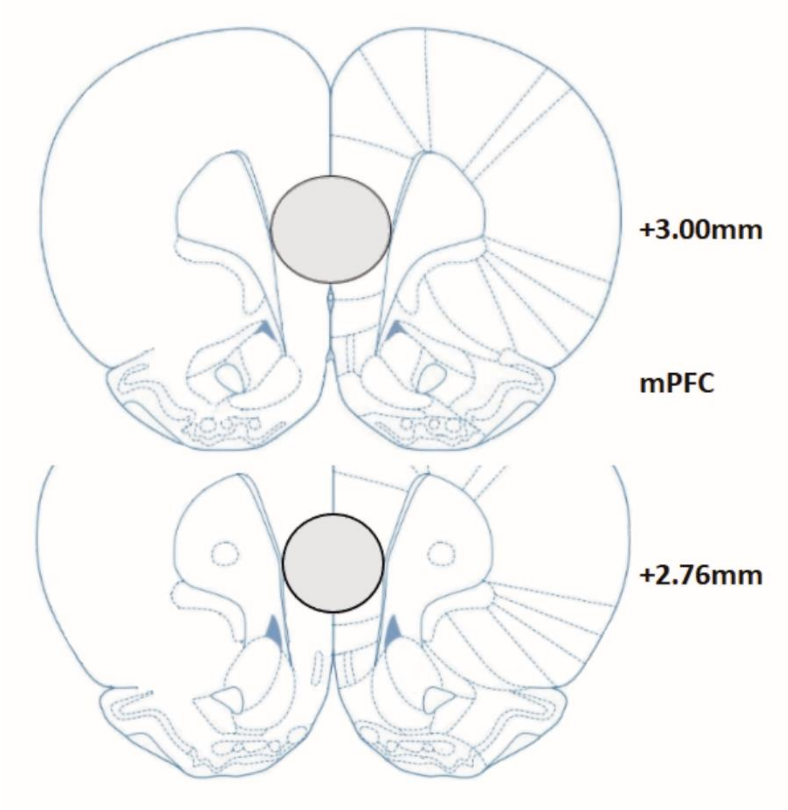

Figure S11: Cannula placements for animals used in the medial prefrontal cortex infusions. All animals' placements were verified post mortem and their data included in the analysis.

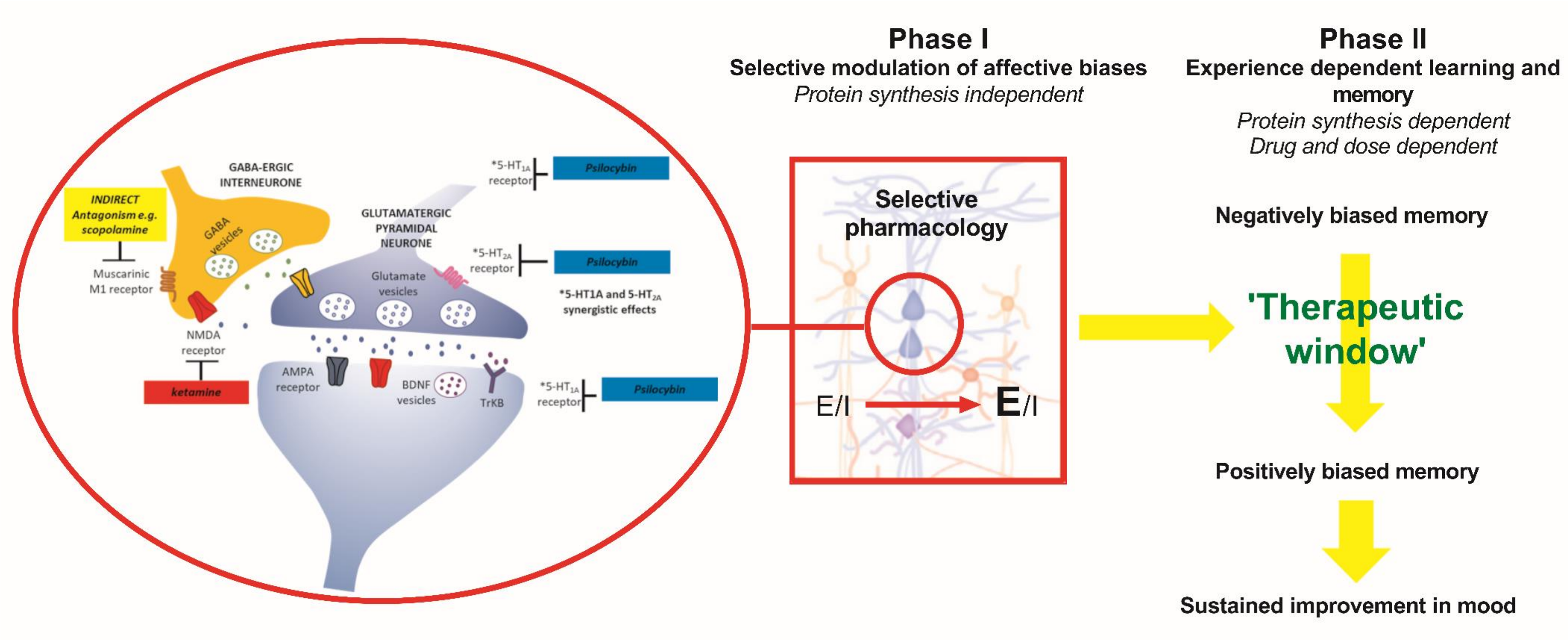

Figure S12: Turning the glass from half empty to half full: interactions between the acute pharmacological effects of RAADs, affective biases, and experience-dependent learning and memory which could explain their rapid and sustained effects on mood. We propose that ketamine, psilocybin and scopolamine act in mPFC to alter glutamate signalling and shift E/I balance generating a 'therapeutic window' where emotional circuits are selectively disengaged or reset to their default mode (PHASE I). During the arising 'therapeutic window', retrieval of memories can occur in the absence of their associated affective bias and, under appropriate conditions, can be re-activated and re-learnt with a relatively more positive affective bias (PHASE II). These findings and this arising hypothesis may provide the missing link between preclinical studies suggesting neuroplastic effects and the rapid and sustained improvements in mood observed in the clinic.

Supplementary Tables

	Substrate 'A'	Substrate 'B'	Substrate 'C'
**test 1**	Felt	shredded dishcloth blue	exfoliating gloves
**test 2**	absorbent fibre	string	foam shapes
**test 3**	Dusters	tissue paper balls	yellow bath sponge
**test 4**	black satin	cardboard	rope
**test 5**	Fur	polyester	pompoms
**test 6**	cellulose sponge	corrugated paper	perlite
**test 7**	purple ribbon	green raffia ribbon	sparkly pompoms
**test 8**	brown pet bedding	cork	hessian sack
**test 9**	cotton wool balls	stringy cloth	hairbands
**test 10**	organza	silk	shredded paper
**test 11**	bin liner	plastic scourer	straws
**test 12**	cotton mix	leather	balloons
**test 13**	chubby wool	shoe laces	velcro
**test 14**	brown partition paper	dishcloth squares	polyester lining
**test 15**	aspen	cypress	coloured matchsticks
**test 16**	Christmas ribbon	umbrella	tights
**test 17**	towel	canvas	pipe cleaners
**test 18**	newspaper	paper pet bedding	confetti
**test 19**	suede	chenille strands	yellow fleece
**test 20**	poster squares	polystyrene	sequins
**test 21**	crepe paper squares	scarf yarn	sparkling fibre
**test 22**	denim	rucksack strap	foam pad

Table S1: List of the substrates used in the experiments in both cohorts.

	Day 1	Day 2	Day 3	Day 4	Day 5/6
	**Pairing 1**	**Pairing 2**	**Pairing 3**	**Pairing 4**	**Choice Test**
Group 1	A vs. C<br>**Drug**	B vs. C<br>**Vehicle**	A vs. C<br>**Drug**	B vs. C<br>**Vehicle**	A vs. B,<br>30 trials
Group 2	B vs. C<br>**Drug**	A vs. C<br>**Vehicle**	B vs. C<br>**Drug**	A vs. C<br>**Vehicle**	A vs. B,<br>30 trials
Group 3	A vs. C<br>**Vehicle**	B vs. C<br>**Drug**	A vs. C<br>**Vehicle**	B vs. C<br>**Drug**	A vs. B,<br>30 trials
Group 4	B vs. C<br>**Vehicle**	A vs. C<br>**Drug**	B vs. C<br>**Vehicle**	A vs. C<br>**Drug**	A vs. B,<br>30 trials

Table S2A : Standard procedure for testing drug-induced affective bias versus vehicle.
Each animal receives drug treatment or vehicle counterbalanced over the four substrate-reward pairing sessions. Substrate (reward-paired substrates - 'A' or 'B' versus unrewarded substrate - 'C') and day are also counter-balanced resulting in four different groups.

	Day 1	Day 2	Day 3	Day 4	Day 5
	**Pairing 1**	**Pairing 2**	**Pairing 3**	**Pairing 4**	**Choice Test**
Group 1	A vs. C<br>**2 pellets**	B vs. C<br>**1 pellet**	A vs. C<br>**2 pellets**	B vs. C<br>**1 pellet**	A vs. B,<br>30 trials
Group 2	B vs. C<br>**2 pellets**	A vs. C<br>**1 pellet**	B vs. C<br>**2 pellets**	A vs. C<br>**1 pellet**	A vs. B,<br>30 trials
Group 3	A vs. C<br>**1 pellet**	B vs. C<br>**2 pellets**	A vs. C<br>**1 pellet**	B vs. C<br>**2 pellets**	A vs. B,<br>30 trials
Group 4	B vs. C<br>**1 pellet**	A vs. C<br>**2 pellets**	B vs. C<br>**1 pellet**	A vs. C<br>**2 pellets**	A vs. B,<br>30 trials

Table S2B : Standard procedure for testing in the reward learning assay.
Each animal receives 2 pellets or 1 pellet counterbalanced over the four substrate-reward pairing sessions. Substrate (reward-paired substrates - 'A' or 'B' versus unrewarded substrate - 'C') and day are also counter-balanced resulting in four different groups.

	Day 1	Day 2	Day 3	Day 4	Day 5	**Day 6**
Group	**Pairing 1**	**Pairing 2**	**Pairing 3**	**Pairing 4**	**Treatment group**	**Choice Test**
**WEEK 1**						
1	A vs. C **FG7142**	B vs. C **Vehicle**	A vs. C **FG7142**	B vs. C **Vehicle**	Ketamine + re-exposure to FG7142 cue	A vs. B, 30 trials
**2**	B vs. C **FG7142**	A vs. C **Vehicle**	B vs. C **FG7142**	A vs. C **Vehicle**	Vehicle $_K$ no re-exposure	A vs. B, 30 trials
3	A vs. C **Vehicle**	B vs. C **FG7142**	A vs. C **Vehicle**	B vs. C **FG7142**	Ketamine + re-exposure to Vehicle $_F$ cue	A vs. B, 30 trials
4	B vs. C **Vehicle**	A vs. C **FG7142**	B vs. C **Vehicle**	A vs. C **FG71422**	Ketamine no re-exposure	A vs. B, 30 trials
**WEEK 2**						
**1**	A vs. C **Vehicle**	B vs. C **FG7142**	A vs. C **Vehicle**	B vs. C **FG7142**	Ketamine no re-exposure	A vs. B, 30 trials
**2**	B vs. C **Vehicle**	A vs. C **FG7142**	B vs. C **Vehicle**	A vs. C **FG7142**	Ketamine + re-exposure to Vehicle $_F$ cue	A vs. B, 30 trials
**3**	A vs. C **FG7142**	B vs. C **Vehicle**	A vs. C **FG7142**	B vs. C **Vehicle**	Vehicle $_K$ no re-exposure	A vs. B, 30 trials
**4**	B vs. C **FG7142**	A vs. C **Vehicle**	B vs. C **FG7142**	A vs. C **Vehicle**	Ketamine + re-exposure to FG7142 cue	A vs. B, 30 trials
**WEEK 3**						
**1**	A vs. C **FG7142**	B vs. C **Vehicle**	A vs. C **FG7142**	B vs. C **Vehicle**	Ketamine + re-exposure to Vehicle $_F$ cue	A vs. B, 30 trials
**2**	B vs. C **FG7142**	A vs. C **Vehicle**	B vs. C **FG7142**	A vs. C **Vehicle**	Ketamine + re-exposure to FG7142 cue	A vs. B, 30 trials
**3**	A vs. C **Vehicle**	B vs. C **FG7142**	A vs. C **Vehicle**	B vs. C **FG7142**	Ketamine no re-exposure	A vs. B, 30 trials
**4**	B vs. C **Vehicle**	A vs. C **FG7142**	B vs. C **Vehicle**	A vs. C **FG7142**	Vehicle $_K$ no re-exposure	A vs. B, 30 trials
**WEEK 4**						
**1**	A vs. C **Vehicle**	B vs. C **FG7142**	A vs. C **Vehicle**	B vs. C **FG7142**	Vehicle $_K$ no re-exposure	A vs. B, 30 trials
**2**	B vs. C **Vehicle**	A vs. C **FG7142**	B vs. C **Vehicle**	A vs. C **FG7142**	Ketamine no re-exposure	A vs. B, 30 trials
**3**	A vs. C **FG7142**	B vs. C **Vehicle**	A vs. C **FG7142**	B vs. C **Vehicle**	Ketamine + re-exposure to FG7142 cue	A vs. B, 30 trials
**4**	B vs. C **FG7142**	A vs. C **Vehicle**	B vs. C **FG7142**	A vs. C **Vehicle**	Ketamine + re-exposure to Vehicle $_F$ cue	A vs. B, 30 trials

Table S2C : Experimental design for the ketamine cue reactivation study.
Each animal receives drug FG7142 or Vehicle $_F$ treatment counterbalanced over the four substrate-reward pairing sessions. Substrate (reward-paired substrates - ‘A’ or ‘B’ versus unrewarded substrate - ‘C’), pairing days and treatment groups are also counter-balanced resulting in four different groups.

Cohort	Treatment	Dose (mg/kg)	Route of administration	Pre-treatment times
**1, 3, 4, 7, 9**<br>**2, 8**	Ketamine	0.0, 1.0, 3.0, 10.0, 25.0<br>0.0, 1.0μg/μl	IP (systemic)<br>mPFC infusion	60min./24hrs<br>60min./24hrs
**2, 8**	Anisomycin	0.0, 100.0μg/μl	mPFC infusion	90min./24.5hrs
**3**	Psilocybin	0.0, 0.1, 0.3, 1.0	IP (systemic)	60min./24hrs
**1, 5, 6**	Scopolamine	0.0, 0.03, 0.1	IP (systemic)	60min./24hrs
**10**	DOI	1. 0μg/μl	mPFC infusion	
**3**	Venlafaxine	0.0, 3.0	IP (systemic)	60min.
**1, 2, 5, 8, 10**	Corticosterone	0.0, 10.0	SC (systemic)	30min.
**3, 9**	FG7142	0.0, 3.0	SC (systemic)	30min.

Table S3: Summary of drug treatments in all cohorts.

Treatment		Response latency (s)		Trials to criterion	
		Vehicle	Drug	Vehicle	Drug
**Psilocybin 0.1-0.3mg/kg and ketamine 1.0mg/kg**	Week 1	2.4±0.2	2.6±0.2	6.6±0.1	6.5±0.2
	Week 2	2.2±0.2	2.0±0.1	6.5±0.1	6.5±0.1
	Week 3	2.0±0.1	2.1±0.1	6.3±0.1	6.2±0.1
	Week 4	2.1±0.2	2.2±0.2	6.6±0.1	6.6±0.1
**Scopolamine 0.1mg/kg**	Week 1	3.0±0.3	2.9±0.3	6.7±0.2	6.7±0.1
	Week 2	2.8±0.2	2.6±0.2	6.4±0.1	6.4±0.1
**Ketamine 10.0-25.0mg/kg**	Week 1	1.4±0.0	1.4±0.0	6.9±0.2	7.3±0.3
	Week 2	1.6±0.0	1.7±0.0	7.1±0.2	7.3±0.3
	Week 3	1.6±0.1	1.6±0.1	7.2±0.2	7.3±0.2
**Psilocybin 1.0mg/kg**	Week 1	1.5±0.0	1.5±0.1	6.3±0.1	6.2±0.1
	Week 2	1.6±0.1	1.7±0.1	6.3±0.1	6.3±0.1

Table S4: Pairing sessions data: number of trials to criterion and latency to dig in the rapid antidepressant effects studies. Data shown as mean (n=11-16 animals/group) ± SEM averaged from the two pairing sessions for each substrate-reward association (vehicle or drug). There were no significant effects during pairing sessions, either on response latency to dig or number of trials to criterion following treatment with vehicle or any of the drugs.

Treatment	Dose (mg/kg)	Response latency (s)
**Vehicle**	0.0	1.8±0.1
**Psilocybin**	0.1	1.7±0.1
**Psilocybin**	0.3	1.6±0.1
**Ketamine**	1.0	1.6±0.1
**Vehicle**	0.0	2.5±0.1
**Scopolamine**	0.1	2.7±0.3
**Vehicle**	0.0	**1.8±0.1**
**Ketamine**	10.0	**5.0±0.7***
**Ketamine**	25.0	**19.6±1.4*****
**Psilocybin**	0.0	1.4±0.0
	1.0	1.4±0.0

Table S5 : Choice bias data: response latency to make choice in the rapid antidepressant effects studies. Data shown as mean (N=11-16 animals/group) ± SEM of an individual latencies during 30 trials of the choice test. No significant difference in latency to make choice was observed in studies following treatment with vehicle or any of the drugs: psilocybin (0.1-0.3mg/kg), scopolamine (0.1mg/kg) and psilocybin (1.0mg/kg). The significant differences were observed in the ketamine (10-25mg/kg) study (RM ANOVA, $F_{2,22}=128.6$, $p<0.0001$, for details see Fig. S1), rats were significantly slower to make a choice following mid (10 mg/kg, $p=0.0249$) and high (25mg/kg, $p<0.0001$) dose of ketamine comparing to vehicle treatment.

	Vehicle		Ketamine 1.0mg/kg		Psilocybin 0.1mg/kg		Psilocybin 0.3mg/kg	
**RAT ID**	Head twitches	Wet dog shakes	Head twitches	Wet dog shakes	Head twitches	Wet dog shakes	Head twitches	Wet dog shakes
**1**	0	0	0	0	0	0	0	1
**2**	0	0	0	0	0	0	0	2
**3**	0	0	0	0	0	0	0	0
**4**	0	0	0	0	0	0	0	0
**5**	0	0	0	0	0	0	0	2
**6**	0	0	0	0	0	0	0	2
**7**	0	0	0	0	0	0	0	3
**8**	0	0	0	0	0	0	0	2
**9**	0	0	0	0	0	0	0	0
**10**	0	0	0	0	0	0	0	0
**11**	0	0	0	0	0	0	0	3
**12**	0	0	0	0	0	0	0	2

Table S6. Choice test data: number of head twitches and wet dog shakes following vehicle and drug treatments in the rapid antidepressant effect study utilising psilocybin 0.1mg/kg and 0.3mg/kg. All drugs were administered 60 min. prior to the choice test. Animals were observed for 15 min. during testing session and the number of head twitches and wet dog shakes were scored.

	Vehicle		Psilocybin 1.0mg/kg	
**RAT ID**	Head twitches	Wet dog shakes	Head twitches	Wet dog shakes
**1**	0	0	2	0
**2**	0	0	3	1
**3**	0	0	0	2
**4**	0	0	2	1
**5**	0	0	0	1
**6**	0	0	2	0
**7**	0	0	2	0
**8**	0	0	0	0
**9**	0	0	1	1
**10**	0	0	0	3
**11**	0	0	1	2
**12**	0	0	2	0

Table S7. Choice test data: number of head twitches and wet dog shakes following vehicle and drug treatments in the rapid antidepressant effect study utilising psilocybin 1.0mg/kg. All drugs were administered 60 min. prior to the choice test. Animals were observed for 15 min. during testing session and the number of head twitches and wet dog shakes were scored.

Treatment	Dose (mg/kg)	Response latency (s)
**Vehicle**	0.0	1.4±0.0
**Psilocybin**	0.1	1.4±0.0
**Psilocybin**	0.3	1.5±0.1
**Psilocybin**	1.0	1.5±0.0
**Vehicle**	0.0	2.1±0.1
**Scopolamine**	0.1	2.2±0.1
**Vehicle**	0.0	1.9±0.1
**Ketamine**	1.0	1.9±0.1
**Vehicle**	0.0	**3.3±0.2**
**Ketamine**	10.0	**4.3±0.4****
**Vehicle**	0.0	**2.9±0.3**
**Ketamine**	25.0	**5.0±0.6****

Table S8: Choice bias data: response latency to make choice in the reward learning assay studies. Data shown as mean (N=11-16 animals/group) ± SEM of an individual latencies during 30 trials of the choice test. No significant difference in latency to make choice was observed in studies following treatment with vehicle or any of the drugs: psilocybin (0.1-1.0mg/kg), scopolamine (0.1mg/kg) and ketamine (1.0mg/kg). The significant differences were observed in the studies with mid (10mg/kg, paired t-test, t_{15},=3.213, p=0.0058) and high dose (25mg/kg, paired t-test, t_{12},=3.835, p=0.0024) of ketamine.

	Vehicle		Psilocybin 0.1mg/kg		Psilocybin 0.3mg/kg		Psilocybin 1.0mg/kg	
**RAT ID**	Head twitches	Wet dog shakes	Head twitches	Wet dog shakes	Head twitches	Wet dog shakes	Head twitches	Wet dog shakes
**1**	0	0	0	0	0	2	2	1
**2**	0	0	0	0	0	2	1	2
**3**	0	0	0	0	0	1	2	0
**4**	0	0	0	0	0	0	1	1
**5**	0	0	0	0	0	1	0	0
**6**	0	0	0	0	0	2	2	1
**7**	0	0	0	0	0	2	1	0
**8**	0	0	0	0	0	1	2	1
**9**	0	0	0	0	0	0	1	1
**10**	0	0	0	0	0	2	2	0
**11**	0	0	0	0	0	2	3	2
**12**	0	0	0	0	0	0	0	2

Table S9. Choice test data: number of head twitches and wet dog shakes following vehicle and drug treatments in the reward learning assay utilising psilocybin 0.1-1.0mg/kg. All drugs were administered 60 min. prior to the choice test. Animals were observed for 15 min. during testing session and the number of head twitches and wet dog shakes were scored.

Treatment	Dose (mg/kg)	Response latency (s)
**Vehicle**	0.0	2.01±0.1
**Ketamine**	1.0	2.08±0.1
**Vehicle**	0	1.4±0.0
**Ketamine**	1.0	1.4±0.1
**Ketamine**	10.0	1.4±0.1
**Ketamine**	25.0	1.5±0.0
**Vehicle**	0.0	1.6±0.0
**Psilocybin**	0.1	1.6±0.0
**Psilocybin**	0.3	1.5±0.0
**Ketamine**	1.0	1.6±0.1
**Vehicle**	0.0	3.8±0.5
**Scopolamine**	0.1	3.2±0.3
**Psilocybin 1.0mg/kg**	0.0	1.5±0.0
	1.0	1.4±0.0

Table S10: Choice bias data: response latency to make choice in the sustained antidepressant effects studies. Data shown as mean (N=11-15 animals/group) ± SEM of an individual latencies during 30 trials of the choice test. No significant difference in latency to make choice was observed in studies following any treatment with vehicle or any of the drugs.

Treatment	Dose (mg/kg)	Response latency (s)		Trials to criterion	
		Vehicle	Drug	Vehicle	Drug
**Vehicle**	0.0	3.9±0.5	3.5±0.7	6.5±0.2	6.7±0.2
**Ketamine**	1.0	4.2±0.4	3.7±0.3	6.7±0.3	7.3±0.4
**Ketamine**	3.0	3.8±0.4	4.5±0.6	6.9±0.3	6.5±0.2
**Ketamine**	10.0	4.0±0.67	5.8±1.2	6.6±0.2	7.0±0.4
**Vehicle**	0.0	2.4±0.2	2.3±0.1	6.8±0.2	6.4±0.1
**Psilocybin**	0.1	2.4±0.1	2.8±0.2	6.5±0.2	6.7±0.2
**Psilocybin**	0.3	2.9±0.3	2.6±0.2	6.8±0.1	6.6±0.2
**Psilocybin**	1.0	**2.4±0.2**	**5.2±0.5*** **	6.4±0.1	6.9±0.1
**Venlafaxine**	3.0	2.4±0.1	2.5±0.1	6.5±0.1	6.5±0.1
**Vehicle**	0.0	3.6 ±0.6	3.8± 0.4	6.6±0.2	6.3±0.2
**Scopolamine**	0.03	3.4±0.4	4.0±0.6	6.8±0.3	6.2±0.1
**Scopolamine**	0.1	**3.7±0.6**	**5.6±1.3***	6.4±0.2	6.3±0.1

Table S11 : Pairing sessions data: number of trials to criterion and latency to dig in the new learning studies. Data shown as mean (N=12-15 animals/group) ± SEM averaged from the two pairing sessions for each substrate-reward association (vehicle or drug). There were no significant effects during pairing sessions, either on response latency to dig or number of trials to criterion following treatment with vehicle or ketamine (1.0-10.0mg/kg). Only treatment with the highest psilocybin dose (1.0mg/kg, paired t-test, t11=7.003, p<0.0001) and the highest scopolamine dose (0.1mg/kg, paired t-test, t11=2.414, p=0.0343) resulted in slower latency to dig comparing to the vehicle group.

	VEHICLE				VENLAFAXINE 3.0mg/kg				PSILOCYBIN 0.1mg/kg				PSILOCYBIN 0.3mg/kg				PSILOCYBIN 1.0mg/kg			
**Rat ID**	head twitches		wet dog shakes		head twitches		wet dog shakes		head twitches		wet dog shakes		head twitches		wet dog shakes		head twitches		wet dog shakes	
**1**	**0**	**0**	**0**	**0**	**0**	**0**	**0**	**0**	**0**	**0**	**0**	**0**	**0**	**0**	**4**	**2**	**0**	**0**	**2**	**0**
**2**	**0**	**0**	**0**	**0**	**0**	**0**	**0**	**0**	**0**	**0**	**0**	**0**	**0**	**0**	**2**	**0**	**1**	**1**	**2**	**0**
**3**	**0**	**0**	**0**	**0**	**0**	**0**	**0**	**0**	**0**	**0**	**0**	**0**	**0**	**0**	**3**	**0**	**2**	**2**	**2**	**0**
**4**	**0**	**0**	**0**	**0**	**0**	**0**	**0**	**0**	**0**	**0**	**0**	**0**	**0**	**0**	**0**	**0**	**2**	**1**	**0**	**0**
**5**	**0**	**0**	**0**	**0**	**0**	**0**	**0**	**0**	**0**	**0**	**0**	**0**	**0**	**0**	**0**	**0**	**3**	**3**	**2**	**4**
**6**	**0**	**0**	**0**	**0**	**0**	**0**	**0**	**0**	**0**	**0**	**0**	**0**	**0**	**0**	**0**	**0**	**2**	**3**	**0**	**0**
**7**	**0**	**0**	**0**	**0**	**0**	**0**	**0**	**0**	**0**	**0**	**0**	**0**	**0**	**0**	**0**	**2**	**1**	**4**	**0**	**3**
**8**	**0**	**0**	**0**	**0**	**0**	**0**	**0**	**0**	**0**	**0**	**0**	**0**	**0**	**0**	**2**	**2**	**2**	**2**	**0**	**0**
**9**	**0**	**0**	**0**	**0**	**0**	**0**	**0**	**0**	**0**	**0**	**0**	**0**	**0**	**0**	**2**	**0**	**3**	**1**	**1**	**0**
**10**	**0**	**0**	**0**	**0**	**0**	**0**	**0**	**0**	**0**	**0**	**0**	**0**	**0**	**0**	**2**	**2**	**2**	**2**	**0**	**0**
**11**	**0**	**0**	**0**	**0**	**0**	**0**	**0**	**0**	**0**	**0**	**0**	**0**	**0**	**0**	**0**	**0**	**2**	**0**	**0**	**0**
**12**	**0**	**0**	**0**	**0**	**0**	**0**	**0**	**0**	**0**	**0**	**0**	**0**	**0**	**0**	**0**	**0**	**2**	**1**	**2**	**0**
	**PS 1**	**PS 2**	**PS 1**	**PS 2**	**PS 1**	**PS 2**	**PS 1**	**PS 2**	**PS 1**	**PS 2**	**PS 1**	**PS 2**	**PS 1**	**PS 2**	**PS 1**	**PS 2**	**PS 1**	**PS 2**	**PS 1**	**PS 2**

Table S12: Pairing sessions data: number of head twitches and wet dog shakes following vehicle and drug treatments in the new learning study. All drugs were administered 60 min. prior to substrate-reward pairing session (PS). Animals were observed for 10 min. during pairing session and the number of head twitches and wet dog shakes were scored.

Treatment	Dose	Response latency (s)
**Ketamine infusion**	0.0ug/ul	2.2±0.1
**Ketamine infusion**	1.0ug/ul	2.1±0.1
**Recall 1h**		
**Vehicle infusion-Vehicle IP**	0.0ug/ul -0.0mg/kg	2.1±0.0
**Vehicle infusion-Ketamine IP**	0.0ug/ul -1.0mg/kg	2.2±0.1
**Anisomycin infusion-Vehicle IP**	100.0ug/ul -0.0mg/kg	2.1±0.0
**Anisomycin infusion-Ketamine IP**	100.0ug/ul -1.0mg/kg	2.2±0.0
**Recall 24hrs**		
**Vehicle infusion-Vehicle IP**	0.0ug/ul -0.0mg/kg	1.9±0.1
**Vehicle infusion-Ketamine IP**	0.0ug/ul -1.0mg/kg	1.9±0.1
**Anisomycin infusion-Vehicle IP**	100.0ug/ul -0.0mg/kg	1.8±0.1
**Anisomycin infusion-Ketamine IP**	100.0ug/ul -1.0mg/kg	1.8±0.0
**Ketamine cue reactivation**		
**Vehicle $_K$ no re-exposure**		2.3±0.8
**Ketamine no re-exposure**		1.5±0.1
**Ketamine + re-exposure to FG7142 cue**		1.5±0.1
**Ketamine + re-exposure to control cue**		1.6±0.1

Table S13: Choice bias data: response latency to make choice in the mechanistic studies. Data shown as mean (N=11-12 animals/group) ± SEM of an individual latencies during 30 trials of the choice test. No significant difference in latency to make choice was observed in studies following any treatment with vehicle or any of the drugs.
